# Comparative efficacy and tolerability of ublituximab vs. other monoclonal antibodies in the treatment of relapsing multiple sclerosis: a systematic review and network meta-analysis of randomized trials

**DOI:** 10.3389/fneur.2024.1479476

**Published:** 2024-12-06

**Authors:** Eoin Moloney, Atefeh Mashayekhi, Sakshi Sharma, Vasileios Kontogiannis, Amir Ansaripour, Wallace Brownlee, David Paling, Mehdi Javanbakht

**Affiliations:** ^1^Optimax Access Ltd, Southampton, United Kingdom; ^2^Optimax Access Ltd, Rotterdam, Netherlands; ^3^Queen Square MS Centre, UCL Queen Square Institute of Neurology, London, United Kingdom; ^4^NIHR University College London Hospitals Biomedical Research Centre, London, United Kingdom; ^5^Department of Neurology, Royal Hallamshire Hospital Sheffield, Sheffield, United Kingdom

**Keywords:** relapsing multiple sclerosis, relapsing-remitting, secondary progressive, monoclonal antibodies, ublituximab, systematic review, network meta-analysis

## Abstract

**Background:**

Relapsing multiple sclerosis (RMS) is a chronic, inflammatory disease of the central nervous system. Ublituximab, an anti-CD20 monoclonal antibody (mAb), is indicated for the treatment of RMS. We performed a systematic literature review (SLR) to identify randomized trials reporting the clinical efficacy and tolerability of ublituximab or comparator disease-modifying therapies (DMTs) for treatment of RMS, and assessed their comparative effects using network meta-analysis (NMA).

**Methods:**

The SLR involved a comprehensive search across various medical databases to identify relevant studies. Included studies were randomized controlled trials (RCTs) of an adult RMS population, focusing on treatment with at least one of ublituximab, alemtuzumab, natalizumab, ocrelizumab, or ofatumumab. For outcomes included in the NMA (annualized relapse rate (ARR), confirmed disability progression (CDP), and treatment discontinuation rate), rate ratios (RR) or hazard ratios (HR), along with their 95% confidence intervals (CIs), were calculated. We performed NMA using a contrast-based random-effects model within a frequentist framework for all outcomes. Ranking probabilities among comparators, and intervention rankings for the NMA, were estimated using surface under the cumulative ranking curve (SUCRA).

**Results:**

We included 15 RCTs in the review. For the ARR outcome, there was no statistically significant difference between ublituximab and the other included mAbs [ofatumumab (RR 1.02 (95% CI 0.64–1.62)), natalizumab (RR 0.99 (0.59–1.65)), alemtuzumab (RR 0.86 (0.51–1.46)), and ocrelizumab (RR 0.75 (0.44–1.28))]. For CDP at 6 months, our results showed no statistically significant difference between ublituximab and the comparator mAbs [ofatumumab (HR 0.97 (0.49–1.92)), natalizumab (HR 1.13 (0.53–2.40)), alemtuzumab (HR 1.25 (0.56–2.81)), and ocrelizumab (HR 1.29 (0.57–2.90))]. For CDP at 3 and 6 months, there was no statistically significant difference between ublituximab and placebo. The all-cause treatment discontinuation rate analysis showed no significant difference between ublituximab and other mAbs, except for alemtuzumab.

**Conclusions:**

Results of this SLR-informed NMA showed that there is no statistically significant difference between ublituximab and the other mAbs in terms of clinical efficacy. Additionally, the findings show that there is no statistically significant difference in discontinuation rates with the exception of the comparison with alemtuzumab, which may be attributed to its unique dosing schedule.

## 1 Introduction

Multiple sclerosis (MS) is a central nervous system disorder that is chronic, inflammatory, demyelinating, and neurodegenerative (1). Epstein-Barr virus appears central to the cause of MS, although other environmental and genetic factors also influence disease susceptibility (2, 3). Although MS can occur at any age, the majority of people are diagnosed between the ages of 20 and 50, with most initially having a relapsing disease course (4).

Relapsing multiple sclerosis (RMS) includes people with relapsing-remitting MS (RRMS) and secondary progressive MS (SPMS) who continue to experience relapses. The underlying causes of MS and the reasons behind its unpredictable course are still poorly understood (4). According to recent statistics from the National MS Society, there are around 1 million people with MS in the United States and 2.5 million patients worldwide. In the UK, it is estimated that there are over 130,000 people with MS and 7,000 people receive new diagnoses every year (5).

Although physical disability is often emphasized in the context of MS, cognitive impairment is also common, affecting up to 65% of patients across all stages of the disease. Cognitive domains most frequently impacted include information processing speed, memory, executive function, and visuospatial abilities (6). Cognitive deficits can occur independently of physical disability, which complicates their identification and recognition (7). The mechanisms behind cognitive impairment are linked to damage in both lesions and normal-appearing white matter, as well as gray matter and immunological changes (6). Research indicates that cognitive impairment can significantly reduce patients' quality of life by affecting physical independence and everyday activities (8).

A number of disease-modifying therapies (DMTs) have been approved by the Food and Drug Administration, the European Medicine Agency, and the Medicines and Healthcare products Regulatory Agency to reduce the chance of relapses and disability progression, but the route of administration, efficacy, tolerability, and safety profile of treatments vary (9).

Traditionally understood as a T cell-mediated disorder (10), MS is influenced significantly by other cells of the immune system, with B cells emerging as critical contributors to its pathogenesis (11). Anti-CD20 monoclonal antibodies (mAbs), which work by selectively depleting CD20-expressing B cells, have been shown to reduce relapses, disability progression, and new magnetic resonance imaging (MRI) lesions. These mAbs offer advantages over other DMTs, including long-lasting pharmacodynamic effects that allow for relatively infrequent dosing (12, 13). Their relative efficacy has been established in comparison with alternative treatments (14).

Anti-CD20 mAbs differ in structure, target epitopes, administration routes, dosing regimens, and methods of B cell depletion, but they share a mechanism of action that effectively reduces inflammatory activity, prevents relapses, and lessens disability in patients with RMS (11). While their safety profile includes concerns like infusion-related reactions and hypogammaglobulinemia, ongoing research aims to improve access through alternative dosing strategies and the development of biosimilars (15). Optimizing clinical use requires a thorough understanding of these therapies' mechanisms, administration routes, comparative efficacy, and safety profiles (11).

Ublituximab is a glycoengineering CD20-directed cytolytic mAb designed to enhance effectiveness in targeting B cells. As part of the anti-CD20 class, which includes treatments like rituximab, ocrelizumab, and ofatumumab, ublituximab selectively depletes CD20-expressing B cells (16). Unlike other CD20-targeted mAbs, ublituximab undergoes a unique glycoengineering process that reduces the presence of fucose in its Fc region. This modification enhances its binding affinity to FcγRIIIa receptors on immune effector cells, leading to increased antibody-dependent cellular cytotoxicity (ADCC). This makes ublituximab more potent in depleting CD20-positive B cells, potentially leading to stronger and more sustained therapeutic effects compared to non-glycoengineered anti-CD20 mAbs (17).

CD20 expressing cells are eliminated by ublituximab through at least three distinct mechanisms, including (i) ADCC, (ii) complement-dependent cytotoxicity, and (iii) antibody-dependent cellular phagocytosis (18). In previous *in-vitro* studies, ublituximab demonstrated 25 to 30 times the antibody-dependent cellular cytolysis potential of other anti-CD20 antibodies (19). In phase II and III trials, ublituximab induced B cell depletion within 24 h (20).

Phase III, double-blind studies of ublituximab show significantly lower annualized relapse rates (ARR) and fewer new gadolinium-enhancing and new T2 lesions on MRI than the comparator (teriflunomide) over 96 weeks (21). The ARR results for ublituximab are particularly notable with rates < 0.10 over 96 weeks (0.08 in ULTIMATE I and 0.09 in ULTIMATE II) (21), reflecting less than one relapse per decade.

Given the established efficacy of mAbs approved for MS (14), it is crucial to assess the comparative effectiveness of newer treatments like ublituximab to help patients, clinicians, and payers make informed treatment decisions. We aimed to assess the efficacy of ublituximab compared to currently-recommended mAbs for RMS (alemtuzumab, natalizumab, ocrelizumab, and ofatumumab), by pooling all relevant studies in a systematic literature review (SLR) and performing network meta-analysis (NMA) across key efficacy outcomes.

## 2 Methods

We followed methodological guidance from the Center for Reviews and Dissemination on best practices for conducting systematic reviews in health care (22). Implementation and reporting of this SLR and NMA followed the recommendations and standards of NICE in the UK, and the preferred reporting items for systematic reviews and meta-analyses (PRISMA) statement (23). All of the data included in the study were fully accessible by the authors.

### 2.1 Search strategy

We searched MEDLINE through PubMed, EMBASE, Cochrane Central Register of Controlled Trials (CENTRAL), and ClinicalTrials.gov (via clinicaltrials.gov/) from inception up until September 2023 [December 2023 for later searches of studies focusing on studies of interferon beta-1a (Rebif^®^)], with an update to the entire review performed in June 2024.

Supplementary searches were conducted to search relevant appraisal data (manufacturer submissions and evidence review/assessment group reports) from previous NICE health technology assessments and to review abstracts from the following congresses for up to 5 years prior to the search date: Americas Committee for Treatment and Research in Multiple Sclerosis; European Committee for Treatment and Research in Multiple Sclerosis; American Academy of Neurology; European Academy of Neurology; Consortium of Multiple Sclerosis Centers; International Multiple Sclerosis Cognition Society.

We also reviewed reference lists from eligible trials and related reviews for additional eligible randomized controlled trials (RCTs). Search strategy details are provided in the Supplementary Tables S1–S11. Records meeting the search criteria were downloaded from databases and imported into Rayyan SLR software, where duplicate records were removed.

### 2.2 Study selection

We included phase III or IV RCTs that: (1) enrolled adult patients (aged ≥18 years) with a definite diagnosis of RMS according to McDonald criteria 2010 (24), had documented MRI of the brain with abnormalities consistent with MS, had ≥ two relapses in the prior 2 years or one relapse in the year prior to screening and/or ≥ one gadolinium-enhancing lesion, (2) included patients defined as having RMS (inclusive of RRMS and relapsing SPMS), highly-active MS, or rapidly evolving severe RMS, and (3) randomized patients to ublituximab, or one of the following treatments for RMS, as an intervention or comparator in the study: alemtuzumab, natalizumab, ocrelizumab, ofatumumab. Additional studies which focused on treatment with either interferon beta-1a (Rebif^®^) or teriflunomide were also included in the SLR as these studies were identified as relevant to creating the network of evidence required to perform indirect treatment comparisons, during prior feasibility assessment.

Included treatments were, therefore, selected on the basis of currently-licensed mAbs, with additional DMTs also included in the search strategies in order to ensure that all evidence required to perform indirect comparisons between the target mAbs was identified. Inclusion of these additional DMTs was based on the network of evidence presented in a previous NMA of DMTs for the treatment of RMS (14). We made a deliberate decision to limit the inclusion of non-mAbs in our analysis to the greatest extent possible, ensuring a focused examination of the comparative efficacy of mAbs.

Studies presented in a language other than English and studies or publications representing animal or experimental studies, economic analyses, editorials, reviews, case-reports or case-series, book chapters, or letters were excluded from this review and meta-analysis. Studies that included a population of patients with clinically isolated syndrome or primary progressive disease were omitted. Additionally, phase II trials were excluded due to the likelihood of including smaller population sizes compared to later-phase studies.

**Figure 1:**
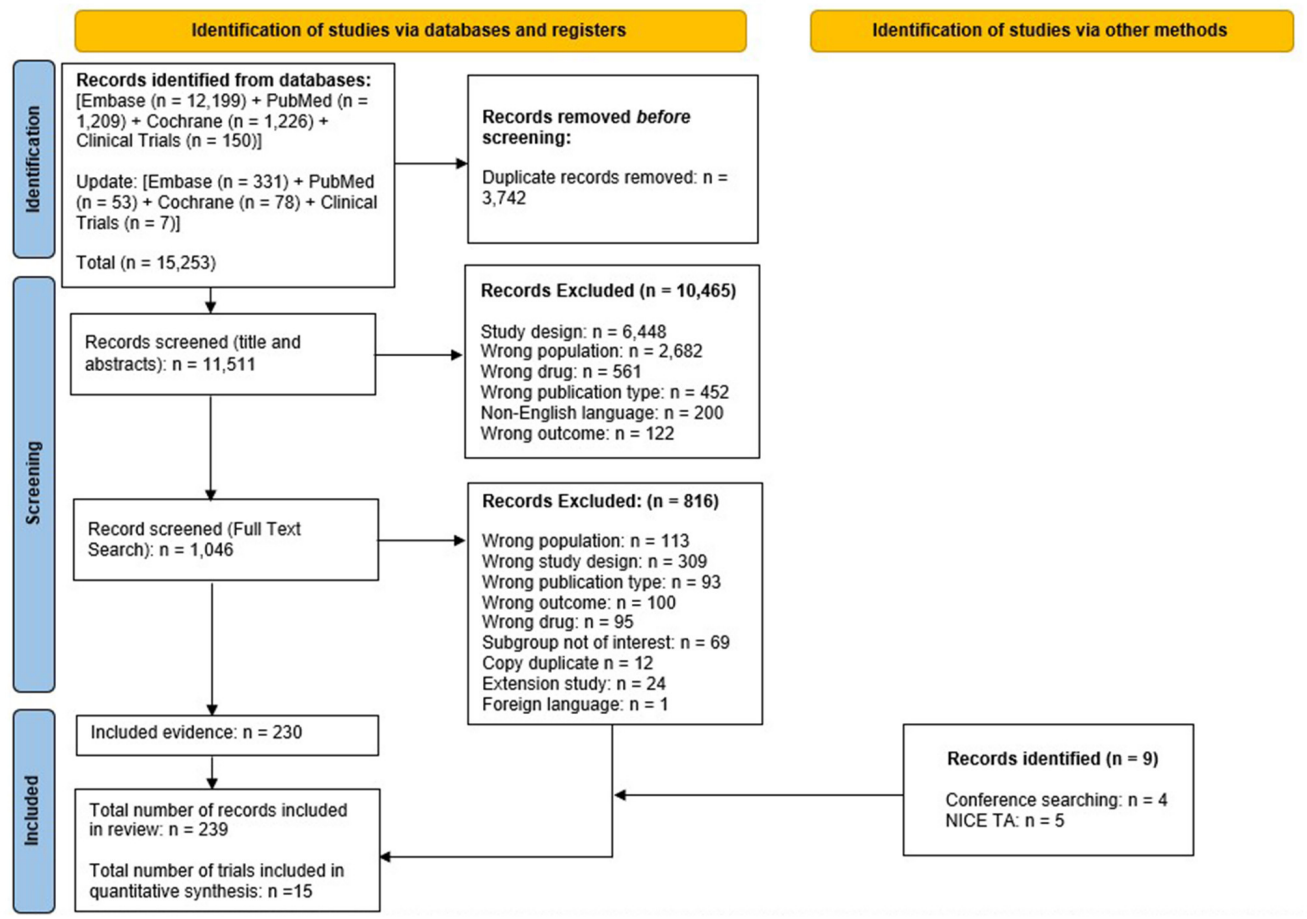
PRISMA flow diagram for the systematic review. NICE TA, national institute for health and care excellence technology appraisal; PRISMA, preferred reporting items for systematic reviews and meta-analyses. From Page et al. (50). For more information, visit. https://www.prisma-statement.org/.

Pairs of reviewers independently screened titles and abstracts of records identified through electronic searches and, subsequently, independently assessed eligibility of those deemed relevant by reviewing their full-text articles. All screening was performed in Rayyan SLR software. Discrepancies were resolved through discussion, or, if needed, by adjudication from a third reviewer. The reasons for exclusion of studies in this phase were logged and reported in the PRISMA flow diagram (Figure 1) (23).

### 2.3 Data extraction

Data from all included studies were extracted using a pre-designed and validated data extraction form developed in Microsoft Excel (Microsoft Corporation, WA, USA). Data extraction was undertaken by one reviewer and checked by a second independent reviewer. Discrepancies were resolved through discussion, or, if needed, by adjudication from a third reviewer.

Extracted data included: study title and year of publication; sponsor and trial identifier; study design, location, and setting; type of intervention and comparators; characteristics of the patient population (including baseline characteristics and details related to patient follow-up and withdrawal); relevant outcomes (outcomes of interest for the presented SLR and NMA were restricted to ARR, confirmed disability progression at 3 (CDP-3m) and 6 months (CDP-6m), and all-cause treatment discontinuation rate); and results reported (including clinical effectiveness and safety of the intervention). Outcome selection for the SLR and NMA was based on the multifaceted nature of MS in terms of its impact on clinical outcomes, and therefore outcomes related to the occurrence of relapses and the progression of disease, as well as discontinuation of treatment, were selected.

Data on outcomes of interest were either extracted from the primary publication associated with a clinical trial or, where results for outcomes of interest were updated with further evidence in a subsequent publication, evidence from the most up-to-date source was extracted and used in the quantitative analysis.

### 2.4 Critical appraisal

The primary publications of clinical studies meeting the criteria for inclusion were assessed by reviewers using an appropriate, and validated, quality assessment instrument, with any disagreements resolved by discussion or following the input of a third reviewer. A complete quality assessment in accordance with the Cochrane Collaboration Risk of Bias for RCTs tool was performed (25).

### 2.5 Data synthesis and statistical methods

The feasibility of performing NMA for each outcome of interest was assessed by checking network connectivity and ensuring the availability of more trials than number of intervention nodes. For all outcomes, we first calculated direct effect estimates by pooling rate ratios (RR) for ARR and all-cause treatment discontinuation, and hazard ratios (HR) for CDP using DerSimonian-Laird random-effects model.

We then performed NMA using a contrast-based random-effects model with a common heterogeneity estimate using the methodology of multivariate meta-analysis using ‘network' suite in Stata (26–28). The 'design-by-treatment' model was used to examine the consistency assumption at network level (global test of consistency). If there was evidence of inconsistency in the network, we used the side-splitting approach to identify if there was a specific modality of interventions that contributed to inconsistency in the network and to run an inconsistency model if we were not able to explain the observed inconsistency. The side-splitting method used to assess local (loop-specific) inconsistency in each closed network loop as the difference between direct and indirect evidence (27, 29).

We visualized the network of interventions using network plots in which the size of the node (circle) corresponds to the number of patients randomized to that intervention and the thickness of the lines corresponds to the number of studies available for each comparison. Comparative effects of interventions for all pairwise comparisons are presented in league tables with placebo as reference intervention.

For the ARR outcome, when studies did not report an annualized rate, we used relapse rate reported for the duration of study and calculated the rate per year for inclusion in the analysis. We performed sensitivity analysis excluding these studies to assess robustness of the results, and also performed a sensitivity analysis based on exclusion of studies where relapse rate had to be imputed based on number of relapse-free patients. For the all-cause treatment discontinuation outcome, we performed a sensitivity analysis excluding the CARE-MS I and II trials due to the unique dosing schedule associated with alemtuzumab.

We also performed network meta-regressions for both the ARR and all-cause treatment outcomes, adjusting for trial follow-up duration. The network meta-regressions on study duration were conducted to explore whether the time at which the outcome was observed (follow-up time) influenced the relative treatment effects.

We estimated ranking probabilities among competing therapies and ranked interventions using surface under the cumulative ranking curve (SUCRA) or mean ranks. Surface under the cumulative ranking curve values are calculated using probability rankings to determine which intervention is most likely to be the most effective—an intervention with a SUCRA value of 100 is considered the most effective, whereas a value of 0 indicates that the intervention is the least effective. Stata (StataCorp., Release 18.0 College Station, TX) (30) was used for all data analyses. All comparisons were two-tailed using a threshold *p*-value ≤ 0.05.

## 3 Results

### 3.1 Systematic review

The systematic search (including updates performed up until June 2024) identified 15,253 studies; from these studies, 3,742 duplicates were removed. The remaining 11,511 studies were screened for eligibility, of which 10,465 studies were excluded based on screening of titles and abstracts. Of 1,046 studies that underwent full-text screening, 816 were deemed ineligible based on exclusion criteria. The remaining 230 studies were included, with an additional nine studies identified via other methods; therefore, 239 records were included in the SLR overall encompassing 15 RCTs in total.

Publications associated with each of the clinical trials, from which data were extracted for inclusion in this SLR and NMA, are referenced accordingly in Table 1. Results of the study selection process are depicted in Figure 1 (23). Details of the critical appraisal of all 15 clinical studies are presented in the Supplementary material, with the majority of studies found to be at low risk of bias, with the notable exceptions of the CARE-MS I and II trials which were found to be of lower overall quality and associated with more risk of bias than other studies included in the NMA, particularly due to their open-label trial design (Supplementary Table S12).

**Table 1:** Summary of clinical studies included in systematic review and network meta-analysis.

**Trial name**	**Location**	**Design**	**No. patients enrolled by study arm**	
			**Intervention(s)**	**Comparator(s)**
AFFIRM (39)	Multinational	RCT (Phase III)	(Natalizumab) 627	(Placebo) 315
ASCLEPIOS I and II (40)	Multinational	RCT (Phase III)	(Ofatumumab) 465 (I), 481 (II)	(Teriflunomide) 462 (I), 474 (II)
CARE-MS I and II (33, 34)	Multinational	RCT (Phase III)	(Alemtuzumab) 386 (I), 436 (II)	(Interferon beta-1a) 195 (I), 231 (II)
IMPROVE (41, 42)	Multinational	RCT (Phase III)	(Interferon beta-1a) 120	(Placebo) 60
OPERA I and II (35)	Multinational	RCT (Phase III)	(Ocrelizumab) 410 (I), 417 (II)	(Interferon beta-1a) 411 (I), 418 (II)
OWIMS (43)	Multinational	RCT (Phase III)	(Interferon beta-1a 22 μg and 44 μg) 95 (22 μg), 98 (44 μg)	(Placebo) 100
PRISMS (36)	Multinational	RCT (Phase III)	(Interferon beta-1a 22 μg and 44 μg) 189 (22 μg), 184 (44 μg)	(Placebo) 187
TEMSO (44)	Multinational	RCT (Phase III)	(Teriflunomide 7 mg and 14 mg) 365 (7 mg), 358 (14 mg)	(Placebo) 363
TENERE (32)	Multinational	RCT (Phase III)	(Teriflunomide 7 mg and 14 mg) 109 (7 mg), 111 (14 mg)	(Interferon beta-1a) 104
TOWER (31)	Multinational	RCT (Phase III)	(Teriflunomide 7 mg and 14 mg) 408 (7 mg), 372 (14 mg)	(Placebo) 389
ULTIMATE I and II (21)	Multinational	RCT (Phase III)	(Ublituximab) 274 (I), 272 (II)	Teriflunomide 275 (I), 273 (II)

RCT, randomized controlled trial.

Table 1 summarizes details of the primary publications associated with the 15 individual RCTs, including information on included comparators and trial names; all records were published from 1998 through 2023. Table 2 presents a brief overview of each RCT.

**Table 2:** Brief overview of clinical studies.

**Trial name**	**Summary**
AFFIRM (39)	Study assessed the efficacy of natalizumab as treatment in participants with RMS, compared to placebo Patients were those who had a diagnosis of RMS; who had a score of 0 to 5 on the EDSS and who had had at least one medically documented relapse within the 12 months before the study began
ASCLEPIOS I and II (40)	Two phase III active-controlled clinical trials of identical design, which assessed the efficacy and safety of subcutaneous ofatumumab as compared with oral teriflunomide Patients were those who had a diagnosis of RMS; who had an EDSS score of 0 to 5.5 and who had at least one relapse in the year before screening, at least two relapses in the 2 years before screening, and a neurologically stable condition for at least 1 month before randomization
CARE-MS I (33)	Study assessed the efficacy and safety of first-line alemtuzumab compared with interferon beta-1a in a phase III trial of patients with previously untreated RRMS (CARE-MS I) Patients were aged 18–50 years and had at least two relapses in the previous 2 years and at least one in the previous year with EDSS scores of 3.0 or lower
CARE-MS II (34)	Study assessed the efficacy and safety of alemtuzumab compared with interferon beta-1a in those who had relapsed while taking first-line treatment Patients were aged 18–55 years with an EDSS score of 5 or less. Patients had at least two relapses in the previous 2 years and at least one in the previous year; at least one relapse while on interferon beta or glatiramer after at least 6 months of treatment
IMPROVE (41, 42)	Study evaluated the efficacy of a new formulation of subcutaneous interferon beta-1a in patients with RRMS Eligible patients were 18–60 years with a diagnosis of RRMS, an EDSS score < 5.5 at study entry, and active disease (1 clinical event and 1 gadolinium-enhancing MRI lesion) within the 6 months before randomization
OPERA I and II (35)	OPERA I and OPERA II investigated the efficacy and safety of ocrelizumab, as compared with subcutaneous interferon beta-1a, in patients with RMS Eligible patients diagnosed with RMS with EDSS score of 0 to 5.5 at screening; at least two clinical relapses within the previous 2 years or one clinical relapse within the year before screening; no neurologic worsening for at least 30 days before both screening and baseline (day 1 trial visit)
OWIMS (43)	Study compared the efficacy of interferon beta-1a, 22 μg or 44 μg weekly, with placebo in patients with RRMS
PRISMS (36)	A double-blind, placebo-controlled study in relapsing/remitting MS investigated the effects of subcutaneous interferon beta-1a compared to placebo Adult patients with relapsing/remitting MS were eligible for study if they had had at least two relapses in the preceding 2 years and had EDSS scores of 0–5.0
TEMSO (44)	Study evaluated the efficacy and safety of teriflunomide in reducing the frequency of relapses and progression of physical disability in patients who had RMS Participants were required to have a score of 5.5 or lower on the EDSS, and at least two clinical relapses in the previous two years or one relapse during the preceding year, but no relapses in the 60 days before randomization
TENERE (32)	Study evaluated the efficacy, safety, and tolerability of teriflunomide compared with interferon beta-1a in patients with RMS Study enrolled patients 18 years of age and older who met McDonald criteria for MS, had a relapsing clinical course with or without progression, and an EDSS score ≤ 5.5 at screening. Patients had to be relapse free for 30 days prior to randomization
TOWER (31)	The phase III TOWER trial assessed the safety and efficacy of teriflunomide in patients with RMS, compared with placebo Patients were aged 18–55 years and had RMS meeting 2005 McDonald criteria, with or without underlying progression, an EDSS score of 5.5 points or less, at least one relapse in the previous year or at least two relapses in the previous 2 years, and no relapse in the 30 days before randomization
ULTIMATE I and II (21)	ULTIMATE I and ULTIMATE II studies evaluated the efficacy and safety of ublituximab infusions as compared with oral teriflunomide, an inhibitor of pyrimidine synthesis, in patients with RMS Eligible patients were aged 18–55 years; were diagnosed with RMS, at least two relapses in the previous 2 years, or one relapse or at least one gadolinium-enhancing lesion or both in the year before screening; EDSS score of 0 to 5.5 at screening and neurologic stability for at least 30 days before screening and the baseline assessment

EDSS, expanded disability status scale; MRI, magnetic resonance imaging; MS, multiple sclerosis; RMS, relapsing multiple sclerosis; RRMS, relapsing-remitting multiple sclerosis.

Treatment comparisons presented in the identified RCTs included natalizumab vs. placebo (AFFIRM), ofatumumab vs. teriflunomide (ASCLEPIOS I and II), alemtuzumab vs. interferon beta-1a (CARE-MS I and II), interferon beta-1a vs. placebo (IMPROVE), interferon beta-1a 22 μg vs. interferon beta-1a 44 μg vs. placebo (OWIMS and PRISMS), ocrelizumab vs. interferon beta-1a (OPERA I and II), teriflunomide 7 mg vs. teriflunomide 14 mg vs. placebo (TEMSO and TOWER), teriflunomide 7 mg vs. teriflunomide 14 mg vs. interferon beta-1a (TENERE), and ublituximab vs. teriflunomide (ULTIMATE I and II).

Table 3 summarizes baseline patient characteristics. Patients had an average age of between 30 and 40 across trials, while the majority of patients were female in most studies. Ethnicity was predominantly white in the majority of studies which reported this information, ranging from 81% in a single treatment arm of the TOWER study at the lower end, to 100% of patients in the TENERE study (31, 32). Black and Asian were the next most prominent ethnicities across included studies.

**Table 3:** Baseline clinical characteristics across included studies.

**Clinical study**	**Study arm**	**Age (years)**	**Gender (male, %)**	**Time since symptom onset—years**	**Time since diagnosis—years**	**EDSS score**	**No. relapses in previous 12 months (mean per patient)**	**No. patients with Gd+ T1 lesions**
AFFIRM (39)	Natalizumab	35.6 ± 8.5	28	NR	5.0 (median)	2.3 ± 1.2	1.53 ± 0.91	319
	Placebo	36.7 ± 7.8	33	NR	6.0 (median)	2.3 ± 1.2	1.50 ± 0.77	143
ASCLEPIOS I (40, 45)	Ofatumumab	38.9 ± 8.8	32	8.4 ± 6.8	5.8 ± 6.1	3.0 ± 1.4	1.20 ± 0.60	174
	Teriflunomide	37.8 ± 9.0	31	8.2 ± 7.2	5.6 ± 6.2	2.9 ± 1.4	1.30 ± 0.70	169
ASCLEPIOS II (40, 46)	Ofatumumab	38.0 ± 9.3	34	8.2 ± 7.4	5.6 ± 6.4	2.9 ± 1.3	1.30 ± 0.70	211
	Teriflunomide	38.2 ± 9.5	33	8.2 ± 7.4	5.5 ± 6.0	2.9 ± 1.4	1.30 ± 0.70	183
CARE-MS I (33, 47)	Alemtuzumab	33.0 ± 8.0	36	2.1 ± 1.4	NR	2.0 ± 0.8	145 (overall)	171
	Interferon beta-1a	33.2 ± 8.5	35	2.0 ± 1.3	NR	2.0 ± 0.8	66 (overall)	94
CARE-MS II (34, 48)	Alemtuzumab	34.8 ± 8.4	34	4.5 ± 2.7	NR	2.7 ± 1.3	211 (overall)	178
	Interferon beta-1a	35.8 ± 8.8	35	4.7 ± 2.9	NR	2.7 ± 1.2	107 (overall)	87
IMPROVE (41, 42)	Interferon beta-1a	34.0 ± 7.8	70	NR	NR	2.50 (median)	NR	2.34 (mean per patient)
	Placebo	35.2 ± 10.5	30	NR	NR	2.25 (median)	NR	3.02 (mean per patient)
OPERA I (35)	Ocrelizumab	37.1 ± 9.3	34	6.7 ± 6.4	3.8 ± 4.8	2.9 ± 1.2	1.31 ± 0.65	172
	Interferon beta-1a	36.9 ± 9.3	34	6.3 ± 6.0	3.7 ± 4.6	2.8 ± 1.3	1.33 ± 0.64	155
OPERA II (35)	Ocrelizumab	37.2 ± 9.1	35	6.7 ± 6.1	4.2 ± 5.0	2.8 ± 1.3	1.32 ± 0.69	161
	Interferon beta-1a	37.4 ± 9.0	33	6.7 ± 6.1	4.1 ± 5.1	2.8 ± 1.4	1.34 ± 0.73	172
OWIMS (43)	Interferon beta-1a 22 μg	35.4 ± 7.3	27	6.9 ± 5.1	NR	2.7 ± 1.2	NR	NR
	Interferon beta-1a 44 μg	35.5 ± 7.4	29	6.7 ± 5.3	NR	2.6 ± 1.4	NR	NR
	Placebo	34.9 ± 7.8	26	6.3 ± 4.7	NR	2.6 ± 1.3	NR	NR
PRISMS (36)	Interferon beta-1a 22 μg	34.8 (median)	33	NR	5.4 (median)	2.5 ± 1.2	NR	NR
	Interferon beta-1a 44 μg	35.6 (median)	34	NR	6.4 (median)	2.5 ± 1.3	NR	NR
	Placebo	34.6	25	NR	4.3 (median)	2.4 ± 1.2	NR	NR
TEMSO (44, 49)	Teriflunomide 7 mg	37.4 ± 9.0	30	8.8 ± 6.8	5.3 ± 5.4	2.7 ± 1.3	1.40 ± 0.70	127
	Teriflunomide 14 mg	37.8 ± 8.2	29	8.7 ± 6.7	5.6 ± 5.4	2.7 ± 1.2	1.30 ± 0.70	125
	Placebo	38.4 ± 9.0	24	8.6 ± 7.1	5.1 ± 5.6	2.7 ± 1.3	1.40 ± 0.70	137
TENERE (32)	Teriflunomide 7 mg	35.2 ± 9.2	36	7.0 ± 6.9	NR	2.0 ± 1.2	1.30 ± 0.80	NR
	Teriflunomide 14 mg	36.8 ± 10.3	30	6.6 ± 7.6	NR	2.3 ± 1.4	1.40 ± 0.80	NR
	Placebo	37.0 ± 10.6	32	7.7 ± 7.6	NR	2.0 ± 1.2	1.20 ± 1.00	NR
TOWER (31)	Teriflunomide 7 mg	37.4 ± 9.4	26	8.2 ± 6.8	5.3 ± 5.5	2.7 ± 1.4	1.40 ± 0.70	NR
	Teriflunomide 14 mg	38.2 ± 9.4	31	8.2 ± 6.7	5.3 ± 5.9	2.7 ± 1.4	1.40 ± 0.70	NR
	Placebo	38.1 ± 9.1	30	7.6 ± 6.7	4.9 ± 5.7	2.7 ± 1.4	1.40 ± 0.80	NR
ULTIMATE I (21)	Ublituximab	36.2 ± 8.2	39	7.5 ± 6.5	4.9 ± 5.2	3.0 ± 1.2	1.30 ± 0.60	117
	Teriflunomide	37.0 ± 9.6	35	6.8 ± 5.9	4.5 ± 5.0	2.9 ± 1.2	1.40 ± 0.70	116
ULTIMATE II (21)	Ublituximab	34.5 ± 8.8	35	7.3 ± 6.5	5.0 ± 5.6	2.8 ± 1.3	1.30 ± 0.60	141
	Teriflunomide	36.2 ± 9.0	35	7.4 ± 6.3	5.0 ± 5.2	3.0 ± 1.2	1.20 ± 0.60	135

Data presented are mean ± standard deviation, unless otherwise stated.

EDSS, expanded disability status scale; Gd, gadolinium; NR, not reported.

Time since symptom onset was >6 years in all studies that reported this data, other than in the CARE-MS I and II trials where time since symptom onset was < 3 and < 5 years, respectively (33, 34). Time since diagnosis ranged between 3.7 years in the comparator arm of the OPERA I trial (35), and 6.4 years in the intervention arm of the PRISMS trial (36) (of the studies that reported this data). Baseline expanded disability status scale (EDSS) scores and details of number of relapses experienced in the previous 12 months, were broadly similar across included trials.

#### 3.1.1 Outcomes of interest extracted in systematic review

Input data extracted for the statistical analyses are presented in Supplementary Tables S13–S16.

### 3.2 Network meta-analysis

Due to insufficient data identified across trials to perform subgroup analyses, all outcome analyses described below are based on the overall trial populations.

#### 3.2.1 ARR

The primary analysis included data from 15 RCTs. The network of treatments and number of trials for each direct comparison is shown in Supplementary Figure S1. The analysis showed ublituximab was superior to placebo [RR 0.31 (95% CI: 0.20, 0.47)], and that there was no statistically significant difference between ublituximab and the comparator mAbs, but the results for the comparisons of ublituximab vs. natalizumab, ocrelizumab, and alemtuzumab showed directions in favor of ublituximab. Treatment effect estimates from the NMA are presented in Table 4. Rankings and SUCRA values for this analysis showed ublituximab to be one of the two best treatments (Supplementary Table S17).

**Table 4:** NMA results for ARR outcome (*n* = 15 RCTs).

**Ublituximab**					
0.86 (0.51, 1.46)	**Alemtuzumab**				
0.99 (0.59, 1.65)	1.15 (0.74, 1.77)	**Natalizumab**			
0.75 (0.44, 1.28)	0.87 (0.61, 1.24)	0.76 (0.49, 1.18)	**Ocrelizumab**		
1.02 (0.64, 1.62)	1.18 (0.74, 1.89)	1.03 (0.66, 1.62)	1.35 (0.84, 2.18)	**Ofatumumab**	
**0.31 (0.20, 0.47)**	**0.36 (0.26, 0.50)**	**0.31 (0.24, 0.42)**	**0.41 (0.30, 0.58)**	**0.30 (0.22, 0.43)**	**Placebo**

Results are RR and their 95% CIs. For column compared to row, RR < 1 means the top-left treatment is better (RR >1 favors the treatment in the row). Bolded comparisons are statistically significant. Blue highlighted cells are effect estimates for the comparison of all active drugs vs. placebo.

For the ARR outcome analysis, there was evidence of global inconsistency (*p*-value from design-by-treatment model = 0.002) with five of eight pairwise comparisons showing statistically significant inconsistency from the side-splitting model (Supplementary Table S18). The following sensitivity analyses were performed: (1) an inconsistency model to assess the robustness of the results and account for the observed inconsistency; (2) excluding data from the OWIMS and IMPROVE trials which reported relapse rate results, rather than ARR specifically, due to their shorter trial durations; (3) excluding data from the OWIMS and PRISMS trials where relapse rate had to be imputed based on number of relapse-free patients; (4) a network meta-regression analysis to adjust for varying follow-up durations across included trials.

The results from sensitivity analysis using the inconsistency model were broadly similar to the consistency model, with ublituximab superior to placebo [RR 0.66 (95% CI: 0.58, 0.75)] and no statistically significant difference between ublituximab and the comparator mAbs (Supplementary Tables S19, S20).

The results for sensitivity analysis excluding the OWIMS and IMPROVE trials showed ublituximab was superior to placebo [RR 0.31 (95% CI: 0.21, 0.46)]. There was no statistically significant difference between ublituximab and the other mAbs, but the results for the comparisons of ublituximab vs. alemtuzumab, natalizumab, and ocrelizumab showed directions in favor of ublituximab. Treatment effect estimates from this sensitivity analysis are presented in Supplementary Table S21. Rankings and SUCRA values showed that ublituximab was ranked as one of the two best treatments in this model (Supplementary Table S22).

The results of sensitivity analysis excluding the OWIMS and PRISMS trials were also broadly similar to the base-case analysis (Supplementary Tables S23, S24). Finally, no effect modification was observed in the network meta-regression analysis adjusting for follow-up duration of included trials (Supplementary Table S25).

#### 3.2.2 CDP-3m

Data regarding disability progression at 3 months were reported in 10 RCTs. The network of treatments and number of trials for each direct comparison is provided in Supplementary Figure S2. For this analysis, there was no closed loop of evidence, and the model was assumed consistent by definition.

The analysis showed no evidence of a statistically significant difference between ublituximab and any of the comparator mAbs or placebo, but the results for the comparison of ublituximab vs. placebo showed a direction in favor of ublituximab [HR 0.58 (95% CI: 0.33, 1.03)]. In this analysis, the comparator mAbs did show statistical superiority compared to placebo. Treatment effect estimates from the NMA are presented in Table 5, while rankings and SUCRA values are presented in Supplementary Table S26.

**Table 5:** NMA results for CDP-3m outcome (*n* = 10 RCTs).

**Ublituximab**				
1.00 (0.53, 1.91)	**Natalizumab**			
1.55 (0.74, 3.27)	1.55 (0.89, 2.71)	**Ocrelizumab**		
1.28 (0.72, 2.30)	1.28 (0.80, 2.04)	0.83 (0.45, 1.50)	**Ofatumumab**	
0.58 (0.33, 1.03)	**0.58 (0.43, 0.78)**	**0.37 (0.23, 0.60)**	**0.45 (0.31, 0.65)**	**Placebo**

Results are HR and their 95% CIs. For column compared to row, HR < 1 means the top-left treatment is better (HR >1 favors the treatment in the row). Bolded comparisons are statistically significant. Blue highlighted cells are effect estimates for the comparison of all active drugs vs. placebo.

#### 3.2.3 CDP-6m

Data regarding disability progression at 6 months were reported in 12 RCTs. The network of treatments and number of trials for each direct comparison is provided in Supplementary Figure S3. There was no closed loop of evidence, and the model was assumed consistent by definition.

The analysis showed that there was no statistically significant difference between ublituximab and any of the comparator mAbs or placebo, but the results for the comparisons of ublituximab vs. placebo [HR 0.52 (95% CI: 0.27, 1.02)] and ofatumumab [HR 0.97 (95% CI: 0.49, 1.92)] showed directions in favor of ublituximab (Table 6). In this analysis, the comparator mAbs did show statistical superiority compared to placebo. Rankings and SUCRA values for this analysis are presented in Supplementary Table S27.

**Table 6:** NMA results for CDP-6m outcome (*n* = 12 RCTs).

**Ublituximab**					
1.25 (0.56, 2.81)	**Alemtuzumab**				
1.13 (0.53, 2.40)	0.90 (0.52, 1.57)	**Natalizumab**			
1.29 (0.57, 2.90)	1.03 (0.64, 1.65)	1.14 (0.65, 1.99)	**Ocrelizumab**		
0.97 (0.49, 1.92)	0.78 (0.42, 1.43)	0.86 (0.50, 1.47)	0.75 (0.41, 1.40)	**Ofatumumab**	
0.52 (0.27, 1.02)	**0.42 (0.27, 0.65)**	**0.46 (0.33, 0.64)**	**0.40 (0.26, 0.63)**	**0.54 (0.35, 0.82)**	**Placebo**

Results are HR and their 95% CIs. For column compared to row, HR < 1 means the top-left treatment is better (HR >1 favors the treatment in the row). Bolded comparisons are statistically significant. Blue highlighted cells are effect estimates for the comparison of all active drugs vs. placebo.

#### 3.2.4 All-cause treatment discontinuation

All-cause treatment discontinuation was reported in 13 RCTs. The network of treatments and number of trials for each direct comparison is provided in Supplementary Figure S4. The analysis showed that there was no statistically significant difference between ublituximab and the other mAbs apart from alemtuzumab, where ublituximab was shown to be inferior [RR 2.20 (95% CI: 1.15, 4.20)]. However, the difference in dosing schedule between these treatments means that this comparison is uncertain and therefore, a sensitivity analysis was performed omitting alemtuzumab from the analysis. Treatment effect estimates from the base-case NMA are presented in Table 7. Rankings and SUCRA values for this analysis are presented in Supplementary Table S28.

**Table 7:** NMA results for all-cause treatment discontinuation outcome (*n* = 13 RCTs).

**Ublituximab**					
**2.20 (1.15, 4.20)**	**Alemtuzumab**				
1.11 (0.62, 1.97)	**0.50 (0.26, 0.96)**	**Natalizumab**			
1.11 (0.61, 2.01)	**0.50 (0.32, 0.79)**	1.00 (0.56, 1.81)	**Ocrelizumab**		
1.16 (0.74, 1.82)	**0.53 (0.30, 0.93)**	1.05 (0.65, 1.69)	1.05 (0.63, 1.73)	**Ofatumumab**	
0.91 (0.59, 1.40)	**0.41 (0.25, 0.70)**	0.82 (0.56, 1.20)	0.82 (0.52, 1.29)	0.78 (0.58, 1.05)	**Placebo**

Results are RR and their 95% CIs. For column compared to row, RR < 1 means the top-left treatment is better (RR >1 favors the treatment in the row). Bolded comparisons are statistically significant. Blue highlighted cells are effect estimates for the comparison of all active drugs vs. placebo.

There was no evidence of global inconsistency (p-value from design-by-treatment model = 0.787) or comparison-specific inconsistency from the side-splitting model (Supplementary Table S29). As mentioned, a sensitivity analysis was also performed for this outcome excluding the CARE-MS I and II trials due to the unique dosing schedule associated with alemtuzumab. This analysis showed no statistically significant difference between ublituximab and any of the other mAbs in the network or placebo. Treatment effect estimates from the NMA are presented in Supplementary Table S30, while rankings and SUCRA values are presented in Supplementary Table S31.

Finally, no effect modification was observed in the network meta-regression analysis adjusting for follow-up duration of included trials (Supplementary Table S32).

## 4 Discussion

This SLR and NMA adds to the evidence base that is currently available related to the comparative effectiveness of mAb treatments for RMS. The study included data from 15 previously-conducted RCTs, following an extensive review of the literature, with robust statistical methods employed to compare the effectiveness of therapies simultaneously.

An NMA was performed to generate comparative efficacy and tolerability data for various mAb treatments, including one of the newest mAbs for this patient population, ublituximab. This analysis aims to provide insights for clinical practice to allow people with MS, clinicians, and payers to make informed decisions regarding choice of treatment.

The NMA demonstrated that ublituximab was in the top two most efficacious treatments for reduction in ARR, and also had the highest probability of being the best at 32.6%. These results are consistent with the available clinical evidence for this treatment, with ublituximab resulting in a significant reduction in ARR < 0.10 in the associated phase III studies (21).

Sensitivity analyses for the ARR outcome using the inconsistency model, and following the omission of clinical trials that reported relapse rate as opposed to annualized rates (OWIMS and IMPROVE), showed broadly similar results, with ublituximab in the top two most effective treatments alongside ofatumumab in the analysis excluding the OWIMS and IMPROVE trials (and with the highest probability of being the best at 33.7%). Base-case results for the ARR outcome analysis highlighted the statistical superiority of ublituximab compared to placebo, with no statistically significant difference between ublituximab and the comparator mAbs shown.

For the CDP-3m outcome, there was no evidence of a statistically significant difference between ublituximab and any of the other mAbs. Similarly, the analysis for the CDP-6m outcome showed that there was no statistically significant difference between ublituximab and any of the comparator mAbs. For both of these analyses, the comparator mAbs did show statistical superiority compared to placebo. Results for ublituximab compared to placebo were not statistically significant, but they did show a direction in favor of ublituximab for both the CDP-3m [HR 0.58 (95% CI: 0.33, 1.03)] and CDP-6m [HR 0.52 (95% CI: 0.27, 1.02)] analyses.

For the all-cause treatment discontinuation outcome, the analysis showed superiority of alemtuzumab over ublituximab, with no evidence of a statistically significant difference between ublituximab and the other mAbs. However, alemtuzumab is provided in a different dosing structure, given in five consecutive days over the first year, and three consecutive days in the second year with retreatment only if required. As this is likely to alter discontinuation rates compared to other included treatments, a sensitivity analysis was also performed for this outcome, excluding the CARE-MS I and II trials. Results of this analysis indicated no statistically significant difference between ublituximab and any of the other mAbs.

The results of this study are comparable to a previously-conducted SLR and NMA of therapies for RMS by Samjoo et al. (14), which also highlighted that treatment with mAb therapies was associated with a significant reduction in ARR compared with placebo and other, non-mAb therapies. This trend was also seen in earlier NMAs which explored the efficacy of DMTs for the treatment of RMS (37, 38).

The Samjoo et al. (14) analysis also indicated that ublituximab ranked among the top three most efficacious treatments for the ARR outcome, alongside ofatumumab and alemtuzumab, which is broadly consistent with the results presented in this analysis. Similarly, their work showed that there was a directionally favorable result for ublituximab in the comparison with placebo for disability progression outcomes (both 3 and 6 months), but that the results were not statistically significant (14). As in our own analysis, the CIs for these results for this comparison (ublituximab vs. placebo for disability progression outcomes) are relatively wide, highlighting the uncertainty that may be present in the analysis.

While the methodology presented in this study is robust, there are certain limitations to the analysis that should be addressed including the relatively low number of RCTs, as well as the limited set of outcomes being assessed. Future research should explore additional endpoints and consider real-world data to further enrich our understanding of mAb effectiveness and facilitate improved RMS treatment decision-making. It was also the case that direct evidence comparing multiple mAbs against one other was limited, and therefore it is possible that the statistical model may have depended excessively on indirect evidence. This over-reliance can result in conclusions that are less robust, as the scarcity of such direct comparison data can lead to broader CIs and less precise estimates. Accurately estimating the variance between studies with this model requires an adequate number of studies. It should also be mentioned that while SUCRA is a useful tool for ranking treatments, its interpretation can be complex and sometimes misleading. It translates multidimensional evidence into a one-dimensional ranking, which may oversimplify the decision-making process and not fully capture the uncertainty in treatment effects.

Finally, we employed a random-effects model to accommodate variability across studies, which necessitates presuming a normal distribution for these effects. Should the actual distribution of random effects deviate from normality, the model could produce misleading outcomes.

A further point to highlight was the heterogeneity and cross-trial differences in definitions of included outcomes, most prevalently related to definition of disability progression. Progression is typically defined based on a required increase in EDSS score from a pre-defined baseline score, however the required increase and defined baseline score can be variable across trials in the area of RMS. This heterogeneity should be considered when interpreting the results.

Additionally, there is a possibility that the clinical trial populations from the studies included in this analysis are not fully representative of RMS patients seen in clinical practice, who tend to be older, have co-morbidities and are often from non-white ethnic backgrounds.

While this NMA focused primarily on physical disability and relapse outcomes, the relative efficacy of the mAbs on cognitive outcomes remains unclear. Although some of the pivotal trials included secondary outcome measures related to cognitive function, such as the Symbol Digit Modalities Test, data on these outcomes were not consistently reported across all studies. As a result, it is difficult to draw firm conclusions regarding the comparative effectiveness of these therapies on cognition. Given the significant impact of cognitive impairment on quality of life and everyday functioning, future studies should more consistently assess cognitive outcomes and explore whether certain mAbs offer superior protection against cognitive decline. This is an important gap in the current evidence that future trials should address to provide a more comprehensive understanding of how these therapies affect both physical and cognitive aspects of MS.

Despite these limitations, the study does, however, represent a valuable addition to the existing body of literature focusing on the comparative effectiveness of mAbs for the treatment of RMS, while our adherence to best practices for conducting and reporting NMAs ensures transparency and reproducibility of our findings. In terms of the results of the NMA, it is noteworthy that despite the differences between pivotal trials, the analysis indicates a relatively similar efficacy across the different mAb treatments. This underscores the utility of NMAs in generating standardized comparisons across trials that otherwise have differing designs and populations.

By synthesizing the available data and creating indirect comparisons, this analysis mitigates some of the challenges posed by trial heterogeneity and enables a more nuanced understanding of the relative efficacy of these agents. In this case, the results suggest that, despite variations in study populations and methodologies, mAbs for RMS demonstrate a consistent improvement in outcomes when compared to placebo. This observation supports the robustness of mAb therapies as a treatment class for RMS, with these agents achieving broadly comparable outcomes, even though direct comparisons across trials are limited. The study also provides valuable insights into the efficacy of newer therapies, such as ublituximab, for key clinical endpoints commonly assessed in MS trials.

## Data Availability

The original contributions presented in the study are included in the article/Supplementary material, further inquiries can be directed to the corresponding author.
