# Supplementary material for: Comparative efficacy and tolerability of ublituximab vs. other monoclonal antibodies in the treatment of relapsing multiple sclerosis: a systematic review and network meta-analysis of randomized trials
Source: Front Neurol. 2024 Dec 6;15:1479476. doi: 10.3389/fneur.2024.1479476 (PMC11659144; doi:10.3389/fneur.2024.1479476)
Supplement: Supplementary file 1 [file Data_Sheet_1.pdf]

SUPPLEMENTAL TABLES

Table S1. Search strategy in Embase and MEDLINE (to 18th September 2023)

	**Database: Embase and MEDLINE <to 18th September 2023>**	**Results**
#1	Exp multiple sclerosis/ OR exp myelitis, transverse/ OR exp neuromyelitis optica/ OR exp demyelinating diseases/ OR exp postvaccinal encephalitis/	338,416
#2	(multiple sclerosis or encephalomyelitis or demyelinating disease or neuromyelitis optica or devic or transverse myelitis or optic neuritis).mp	347,061
#3	(relapsing or relaps* or remit* or relapsing remitting*).mp.	700,799
#4	(RRMS or RMS).mp	55,847
#5	#1 OR #2 OR #3 OR #4	1,087,755
#6	exp Ofatumumab/ OR exp alemtuzumab/ OR exp ocrelizumab/ OR exp Ublituximab/ OR exp natalizumab/ OR exp teriflunomide/	41,774
#7	(Teriflunomide or A 771726 or A77 1726 or A77-1726 or A771726 or aubagio or hmr 1726 or hmr1726).mp	6,145
#8	(Ublituximab or Anti-CD20 Monoclonal Antibody or TG-1101).mp.	4,847
#9	(Ofatumumab or OMB157 or HYMAX-CD20 2F2 or HUMAXCD20-2F2 or Arzerra or GSK1841157 or GSK-1841157).mp	4,961
#10	(alemtuzumab or campath 1h or anti-CD52 or anti CD52).mp	24,017
#11	(natalizumab or antegren or tysabri or ocrelizumab or ocrevus or humani#se anti CD20 antibody).mp	20,572
#12	#6 OR #7 OR #8 OR #9 OR #10 OR #11	50,990
#13	#5 AND #12	28,102
#14	exp clinical trial/	2,819,342
#15	exp randomization/ or exp randomized controlled trial/ or exp "randomized controlled trial (topic)"/	1,805,749
#16	exp controlled clinical trial/ or exp "controlled clinical trial (topic)"/	1,938,938
#17	exp clinical trials as topic/	829,394

#18	exp placebo/ or exp placebo effect/	412,053
#19	clinical trial*.mp.	3,215,999
#20	control?ed clinical trial.mp.	615,191
#21	randomi#ed controlled trial.mp.	1,727,458
#22	randomi#ation.mp.	200,879
#23	((random* adj2 allocat*) or (random* adj2 assign*)).tw.	416,188
#24	placebo*.mp.	789,113
#25	(rat or rats or mouse or mice or swine or porcine or murine or sheep or lambs or pigs or piglets or rabbit or rabbits or cat or cats or dog or dogs or cattle or bovine or monkey or monkeys or trout or marmoset$1).ti. and animal experiment/	1,222,524
#26	Animal experiment/ not (human experiment/ or human/)	2,569,898
#27	14 or 15 or 16 or 17 or 18 or 19 or 20 or 21 or 22 or 23 or 24	4,750,145
#28	25 or 26	2,638,451
#29	27 not 28	4,675,680
#30	13 and 29	10,483

Table S2. Search strategy in Embase and MEDLINE – additional searches for interferon beta-1a (to 28th December 2023)

	**Database: Embase and MEDLINE <to 28th December 2023>**	**Results**
#1	Exp multiple sclerosis/ OR exp myelitis, transverse/ OR exp neuromyelitis optica/ OR exp demyelinating diseases/ OR exp postvaccinal encephalitis/	226,973
#2	(multiple sclerosis or encephalomyelitis or demyelinating disease or neuromyelitis optica or devic or transverse myelitis or optic neuritis).mp	220,778
#3	(relapsing or relaps* or remit* or relapsing remitting*).mp.	472,467
#4	(RRMS or RMS).mp	33,668
#5	#1 OR #2 OR #3 OR #4	710,151
#6	exp Interferon-beta/	383,320
#7	(avonex or rebif).mp.	3,752

#8	((interferon or IFN) adj2 (beta 1a or beta-1a or beta1a)).mp.	9,974
#9	(interferon adj2 beta).mp.	43,129
#10	(ifn adj2 beta).mp.	15,477
#11	(IFN beta* or IFNbeta*).mp.	15,655
#12	#6 OR #7 OR #8 OR #9 OR #10 OR #11	392,978
#13	#5 AND #12	29,207
#14	exp clinical trial/	1,869,242
#15	exp randomization/ or exp randomized controlled trial/ or exp "randomized controlled trial (topic)"/	1,135,360
#16	exp controlled clinical trial/ or exp "controlled clinical trial (topic)"/	1,267,682
#17	exp clinical trials as topic/	452,326
#18	exp placebo/ or exp placebo effect/	411,200
#19	clinical trial*.mp.	2,063,262
#20	control?ed clinical trial.mp.	502,398
#21	randomi#ed controlled trial.mp.	1,090,325
#22	randomi#ation.mp.	151,144
#23	((random* adj2 allocat*) or (random* adj2 assign*)).tw.	235,710
#24	placebo*.mp.	530,808
#25	(rat or rats or mouse or mice or swine or porcine or murine or sheep or lambs or pigs or piglets or rabbit or rabbits or cat or cats or dog or dogs or cattle or bovine or monkey or monkeys or trout or marmoset$1).ti. and animal experiment/	1,234,934
#26	Animal experiment/ not (human experiment/ or human/)	2,594,354
#27	14 or 15 or 16 or 17 or 18 or 19 or 20 or 21 or 22 or 23 or 24	2,992,908
#28	25 or 26	2,664,865
#29	27 not 28	2,917,325
#30	13 and 29	8,889
#31	(PRISMS or prisms trial).mp. [mp=title, abstract, heading word, drug trade name, original title, device manufacturer, drug manufacturer, device trade name, keyword heading word, floating subheading word, candidate term word]	3,070
#32	(Avonex or Rebif).mp. [mp=title, abstract, heading word, drug trade name, original title, device manufacturer, drug manufacturer, device trade name, keyword heading word, floating subheading word, candidate term word]	3,752
#33	#31 or #32	6,803

#34	30 and 33	1,716

Table S3. Search strategy in Embase – Update (18th September 2023 to 3rd June 2024)

	Database: Embase <September 2023 to June 2024>	Results
#1	Exp multiple sclerosis/ OR exp myelitis, transverse/ OR exp neuromyelitis optica/ OR exp demyelinating diseases/ OR exp postvaccinal encephalitis/	3,961
#2	(multiple sclerosis or encephalomyelitis or demyelinating disease or neuromyelitis optica or devic or transverse myelitis or optic neuritis).mp	3,413
#3	(relapsing or relaps* or remit* or relapsing remitting*).mp.	12,725
#4	(RRMS or RMS).mp	872
#5	#1 OR #2 OR #3 OR #4	16,226
#6	exp Ofatumumab/ OR exp alemtuzumab/ OR exp ocrelizumab/ OR exp Ublituximab/ OR exp natalizumab/ OR exp teriflunomide/	615
#7	(Teriflunomide or A 771726 or A77 1726 or A77-1726 or A771726 or aubagio or hmr 1726 or hmr1726).mp	71
#8	(Ublituximab or Anti-CD20 Monoclonal Antibody or TG-1101).mp.	115
#9	(Ofatumumab or OMB157 or HYMAX-CD20 2F2 or HUMAXCD20-2F2 or Arzerra or GSK1841157 or GSK-1841157).mp	91
#10	(alemtuzumab or campath 1h or anti-CD52 or anti CD52).mp	244
#11	(natalizumab or antegren or tysabri or ocrelizumab or ocrevus or humani#se anti CD20 antibody).mp	318
#12	#6 OR #7 OR #8 OR #9 OR #10 OR #11	715
#13	#5 AND #12	476
#14	exp clinical trial/	41,891
#15	exp randomization/ or exp randomized controlled trial/ or exp "randomized controlled trial (topic)"/	20,657
#16	exp controlled clinical trial/ or exp "controlled clinical trial (topic)"/	21,105
#17	exp clinical trials as topic/	1,593
#18	exp placebo/ or exp placebo effect/	5,582
#19	clinical trial*.mp.	30,694
#20	control?ed clinical trial.mp.	961
#21	randomi#ed controlled trial.mp.	20,601
#22	randomi#ation.mp.	2,617
#23	((random* adj2 allocat*) or (random* adj2 assign*)).tw.	3,089
#24	placebo*.mp.	5,752

#25	(rat or rats or mouse or mice or swine or porcine or murine or sheep or lambs or pigs or piglets or rabbit or rabbits or cat or cats or dog or dogs or cattle or bovine or monkey or monkeys or trout or marmoset$1).ti. and animal experiment/	11,364
#26	Animal experiment/ not (human experiment/ or human/)	21,387
#27	14 or 15 or 16 or 17 or 18 or 19 or 20 or 21 or 22 or 23 or 24	52,766
#28	25 or 26	24,297
#29	27 not 28	51,736
#30	13 and 29	241

Table S4. Search strategy in MEDLINE – Update (18th September 2023 to 3rd June 2024)

	**Database: MEDLINE <September 2023 to June 2024>**	**Results**
#1	Exp multiple sclerosis/ OR exp myelitis, transverse/ OR exp neuromyelitis optica/ OR exp demyelinating diseases/ OR exp postvaccinal encephalitis/	2,710
#2	(multiple sclerosis or encephalomyelitis or demyelinating disease or neuromyelitis optica or devic or transverse myelitis or optic neuritis).mp	4,605
#3	(relapsing or relaps* or remit* or relapsing remitting*).mp.	9,418
#4	(RRMS or RMS).mp	959
#5	#1 OR #2 OR #3 OR #4	14,136
#6	exp Ofatumumab/ OR exp alemtuzumab/ OR exp ocrelizumab/ OR exp Ublituximab/ OR exp natalizumab/ OR exp teriflunomide/	97
#7	(Teriflunomide or A 771726 or A77 1726 or A77-1726 or A771726 or aubagio or hmr 1726 or hmr1726).mp	79
#8	(Ublituximab or Anti-CD20 Monoclonal Antibody or TG-1101).mp.	75
#9	(Ofatumumab or OMB157 or HYMAX-CD20 2F2 or HUMAXCD20-2F2 or Arzerra or GSK1841157 or GSK-1841157).mp	54
#10	(alemtuzumab or campath 1h or anti-CD52 or anti CD52).mp	92
#11	(natalizumab or antegren or tysabri or ocrelizumab or ocrevus or humani#se anti CD20 antibody).mp	220
#12	#6 OR #7 OR #8 OR #9 OR #10 OR #11	412
#13	#5 AND #12	308
#14	exp clinical trial/	16,730
#15	exp randomization/ or exp randomized controlled trial/ or exp "randomized controlled trial (topic)"/	14,522
#16	exp controlled clinical trial/ or exp "controlled clinical trial (topic)"/	14,371
#17	exp clinical trials as topic/	7,354

#18	exp placebo/ or exp placebo effect/	81
#19	clinical trial*.mp.	33,132
#20	control?ed clinical trial.mp.	1,194
#21	randomi#ed controlled trial.mp.	20,331
#22	randomi#ation.mp.	5,741
#23	((random* adj2 allocat*) or (random* adj2 assign*)).tw.	8,631
#24	placebo*.mp.	7,591
#25	(rat or rats or mouse or mice or swine or porcine or murine or sheep or lambs or pigs or piglets or rabbit or rabbits or cat or cats or dog or dogs or cattle or bovine or monkey or monkeys or trout or marmoset$1).ti.	36,748
#26	exp animals/ not humans.sh	68,619
#27	14 or 15 or 16 or 17 or 18 or 19 or 20 or 21 or 22 or 23 or 24	58,351
#28	25 or 26	87,946
#29	27 not 28	55,643
#30	13 and 29	90

Table S5. Search strategy in PubMed (to 18th September 2023)

	**Database: PubMed <to 18th September 2023>**	**Results**
#1	Multiple Sclerosis/ OR Multiple Sclerosis, Relapsing-Remitting/ OR Myelitis, Transverse/ OR Demyelinating Diseases/ OR Encephalomyelitis, Acute Disseminated/	154,267
#2	(multiple sclerosis or encephalomyelitis or demyelinating disease or neuromyelitis optica or devic or transverse myelitis or optic neuritis).mp	722
#3	#1 OR #2	154,298
#4	("Ofatumumab" OR "Ocrelizumab" OR "Alemtuzumab" OR "Natalizumab" OR "Teriflunomide" OR "Ocrevus" OR "Arzerra" OR "Tysabri" OR "HMR1726" OR "Ublituximab" OR "Briumvi")	8,421
#5	((((randomized controlled trial[pt] OR controlled clinical trial[pt] OR randomized[tiab] OR randomised[tiab] OR placebo[tiab] OR clinical trials as topic[tiab] OR randomly[tiab] OR trial[ti]) OR ((RCT[tiab] OR placebo[tiab] OR sham[tiab] OR dummy[tiab] OR single blind*[tiab] OR double blind*[tiab] OR allocated[tiab] OR allocation[tiab] OR triple blind*[tiab] OR treble blind*[tiab] OR random*[tiab]) NOT (medline[sb])))))	1,562,732

	**Database: PubMed <to 18th September 2023>**	**Results**
#6	#3 AND #4	4,483
#7	#6 AND #5	519

Table S6. Search strategy in PubMed – additional searches for interferon beta-1a (to 28th December 2023)

	**Database: PubMed <to 28th December 2023>**	**Results**
#1	Multiple Sclerosis/ OR Multiple Sclerosis, Relapsing-Remitting/ OR Myelitis, Transverse/ OR Demyelinating Diseases/ OR Encephalomyelitis, Acute Disseminated/	156,347
#2	(multiple sclerosis or encephalomyelitis or demyelinating disease or neuromyelitis optica or devic or transverse myelitis or optic neuritis).mp	746
#3	((((randomized controlled trial[pt] OR controlled clinical trial[pt] OR randomized[tiab] OR randomised[tiab] OR placebo[tiab] OR clinical trials as topic[tiab] OR randomly[tiab] OR trial[ti]) OR ((RCT[tiab] OR placebo[tiab] OR sham[tiab] OR dummy[tiab] OR single blind*[tiab] OR double blind*[tiab] OR allocated[tiab] OR allocation[tiab] OR triple blind*[tiab] OR treble blind*[tiab] OR random*[tiab]) NOT (medline[sb])))))	1,589,003
#4	#1 OR #2	156,379
#5	#3 AND #4	8,170
#6	("PRISMS" OR "prisms trial") OR ("interferon-β-1a" OR "Avonex" OR "Rebif")	19,302
#7	#6 AND #5	690

Table S7. Search strategy in PubMed – Update (18th September 2023 to 3rd June 2024)

	**Database: PubMed <September 2023 to June 2024>**	**Results**
#1	Multiple Sclerosis/ OR Multiple Sclerosis, Relapsing-Remitting/ OR Myelitis, Transverse/ OR Demyelinating Diseases/ OR Encephalomyelitis, Acute Disseminated/	159,513
#2	(multiple sclerosis or encephalomyelitis or demyelinating disease or neuromyelitis optica or devic or transverse myelitis or optic neuritis).mp	777
#3	#1 OR #2	159,545

	**Database: PubMed <September 2023 to June 2024>**	**Results**
#4	("Ofatumumab" OR "Ocrelizumab" OR "Alemtuzumab" OR "Natalizumab" OR "Teriflunomide" OR "Ocrevus" OR "Arzerra" OR "Tysabri" OR "HMR1726" OR "Ublituximab" OR "Briumvi")	8,790
#5	("PRISMS" OR "prisms trial") OR ("interferon-β-1a" OR "Avonex" OR "Rebif")	20,126
#6	#4 OR #5	28,565
#7	((((randomized controlled trial[pt] OR controlled clinical trial[pt] OR randomized[tiab] OR randomised[tiab] OR placebo[tiab] OR clinical trials as topic[tiab] OR randomly[tiab] OR trial[ti]) OR ((RCT[tiab] OR placebo[tiab] OR sham[tiab] OR dummy[tiab] OR single blind*[tiab] OR double blind*[tiab] OR allocated[tiab] OR allocation[tiab] OR triple blind*[tiab] OR treble blind*[tiab] OR random*[tiab]) NOT (medline[sb])))))	1,629,807
#8	'nrct' OR 'n rct' OR n?rct OR (('non' OR 'not') NEAR/3 ('randomised' OR 'randomized')) OR 'controlled clinical trial' OR 'controlled trial'	1,083,210
#9	#7 OR #8	1,734,007
#10	#3 AND #6 AND #9	1,287
#11	#3 AND #6 AND #9 Filters: from 2023/9/14 - 2024/6/3	53

Table S8. Search strategy in Cochrane Library (to 18th September 2023)

	**Database: Cochrane library [Central] <to 18th September 2023>**	**Results**
#1	[Multiple Sclerosis, Relapsing-Remitting] explode all tree	1,132
#2	"Multiple sclerosis" OR "transverse myelitis" OR "optic neuritis" OR "devic" OR "adem" OR "neuromyelitis optica" OR ‘‘Relapse-remitting’’	17,504
#3	#1 OR #2	17,504
#4	‘'Ofatumumab'’ OR ‘'Ocrelizumab'’ OR ‘'Alemtuzumab'’ OR '‘Natalizumab’' OR ‘'Teriflunomide" OR "Ocrevus" OR "Arzerra" OR "Tysabri" OR "HMR1726" OR "MabCambath" OR "Ublituximab" OR "Briumvi"	2,157
#5	#3 AND #4	1,226

Table S9. Search strategy in Cochrane Library – Update (18th September 2023 to 3rd June 2024)

	**Database: Cochrane library [Central] <September 2023 to June 2024>**	**Results**
#1	[Multiple Sclerosis, Relapsing-Remitting] explode all tree	1,300
#2	"Multiple sclerosis" OR "transverse myelitis" OR "optic neuritis" OR "devic" OR "adem" OR "neuromyelitis optica" OR ‘‘Relapse-remitting’’	13,746
#3	#1 OR #2	13,746
#4	‘'Ofatumumab'’ OR ‘'Ocrelizumab'’ OR ‘'Alemtuzumab'’ OR '‘Natalizumab’' OR ‘'Teriflunomide" OR "Ocrevus" OR "Arzerra" OR "Tysabri" OR "HMR1726" OR "MabCambath" OR "Ublituximab" OR "Briumvi"	2,217
#5	(avonex or rebif).mp.	20,919
#6	((interferon or IFN) adj2 (beta 1a or beta-1a or beta1a)).mp.	20,917
#7	#5 OR #6 OR #4	23,060
#8	#3 AND #7	1,692
#9	Filter: sept 2023- June 2024	78

Table S10. Search strategy in ClinicalTrials.gov (to 18th September 2023)

	**Database: ClinicalTrials.gov <to 18th September 2023>**	**Results**
#1	Multiple Sclerosis OR multiple sclerosis OR relapse multiple sclerosis OR demyelinating disease	150

Table S11. Search strategy in ClinicalTrials.gov – Update (18th September 2023 to 3rd June 2024)

	**Database: ClinicalTrials.gov <September 2023 to June 2024>**	**Results**
#1	Multiple Sclerosis OR multiple sclerosis OR relapse multiple sclerosis OR demyelinating disease	7

Table S12. Cochrane Risk of Bias Appraisals

**Randomisation process**	**AFFIRM** (1)	**ASCLEPIOS I & II** (2)	**CARE-MS I** (3)	**CARE-MS II** (4)	**IMPROVE** (5,6)	**OPERA I & II** (7)	**OWIMS** (8)	**PRISMS** (9)	**TEMSO** (10)	**TENERE** (11)	**TOWER** (12)	**ULTIMATE I & II** (13)
1.1 Was the allocation sequence random?	YES	YES	YES	YES	YES	YES	YES	YES	YES	YES	YES	YES
1.2 Was the allocation sequence concealed?	YES	YES	YES	YES	UNCLEAR	YES	YES	YES	UNCLEAR	UNCLEAR	YES	YES
1.3 Did baseline differences between intervention groups suggest a problem with the randomisation process?	NO	NO	NO	YES	NO	NO	NO	NO	NO	YES	NO	NO
**Risk of bias judgement**	LOW	LOW	LOW	LOW	UNCLEAR	LOW	LOW	LOW	UNCLEAR	HIGH	LOW	LOW
**Optional: What is the predicted direction of bias arising from the randomisation process?**	NA	NA	NA	NA	UNCLEAR	NA	NA	NA	UNCLEAR	UNCLEAR	NA	NA
**Deviations from the intended interventions**												
2.1. Were participants aware of their assigned intervention during the trial?	NO	NO	YES	YES	NO	NO	NO	NO	NO	UNCLEAR	NO	NO
2.2. Were carers and people delivering the interventions aware of participants assigned intervention during the trial?	NO	NO	YES	YES	NO	NO	NO	NO	NO	NO	NO	NO
2.3. If Y/PY/NI to 2.1 or 2.2: Were there deviations from the intended intervention that arose because of the trial context?	NA	NA	NO	NO	NA	NA	NA	NA	NA	NO	NA	NA
2.4 If Y/PY to 2.3: Were these deviations likely to have affected the outcome?	NA	NA	NA	NA	NA	NA	NA	NA	NA	NA	NA	NA
2.5. If Y/PY/NI to 2.4: Were these deviations from intended	NA	NA	NA	NA	NA	NA	NA	NA	NA	NA	NA	NA

intervention balanced between groups?												
2.6 Was an appropriate analysis used to estimate the effect of assignment to intervention?	YES	YES	YES	YES	YES	YES	YES	YES	YES	YES	YES	YES
2.7 If N/PN/NI to 2.6: Was there potential for a substantial impact (on the result) of the failure to analyse participants in the group to which they were randomised?	NA	NA	NA	NA	NA	NA	NA	NA	NA	NA	NA	NA
**Risk of bias judgement**	LOW	LOW	HIGH	HIGH	LOW	LOW	LOW	LOW	LOW	LOW	LOW	LOW
**Optional: What is the predicted direction of bias arising from the randomisation process?**	NA	NA	UNCLEAR	UNCLEAR	NA	NA	NA	NA	NA	NA	NA	NA
**Missing outcome data**												
3.1 Were data for this outcome available for all, or nearly all, participants randomised?	YES	YES	YES	YES	YES	YES	YES	YES	YES	YES	YES	YES
3.2 If N/PN/NI to 3.1: Is there evidence that the result was not biased by missing outcome data?	NA	NA	NA	NA	NA	NA	NA	NA	NA	NA	NA	NA
3.3 If N/PN to 3.2: Could missingness in the outcome depend on its true value?	NA	NA	NA	NA	NA	NA	NA	NA	NA	NA	NA	NA
3.4 If Y/PY/NI to 3.3: Is it likely that missingness in the outcome depended on its true value?	NA	NA	NA	NA	NA	NA	NA	NA	NA	NA	NA	NA
**Risk of bias judgement**	LOW	LOW	LOW	LOW	LOW	LOW	LOW	LOW	LOW	LOW	LOW	LOW
**Optional: What is the predicted direction of bias arising from the randomisation process?**	NA	NA	NA	NA	NA	NA	NA	NA	NA	NA	NA	NA
**Measurement of the outcome**												
4.1 Was the method of measuring the outcome inappropriate?	NO	NO	NO	NO	NO	NO	NO	NO	NO	NO	NO	NO

4.2 Could measurement or ascertainment of the outcome have differed between intervention groups?	NO	NO	NO	NO	NO	NO	NO	NO	NO	NO	NO	NO
4.3 If N/PN/NI to 4.1 and 4.2: Were outcome assessors aware of the intervention received by study participants?	NO	NO	NO	NO	UNCLEAR	NO	NO	NO	NO	NO	NO	NO
4.4 If Y/PY/NI to 4.3: Could assessment of the outcome have been influenced by knowledge of intervention received?	NA	NA	NA	NA	NA	NA	NA	NA	NA	NA	NA	NA
4.5 If Y/PY/NI to 4.4: Is it likely that assessment of the outcome was influenced by knowledge of intervention received?	NA	NA	NA	NA	NA	NA	NA	NA	NA	NA	NA	NA
**Risk of bias judgement**	LOW	LOW	LOW	LOW	UNCLEAR	LOW	LOW	LOW	LOW	LOW	LOW	LOW
**Optional: What is the predicted direction of bias arising from the randomisation process?**	NA	NA	NA	NA	UNCLEAR	NA	NA	NA	NA	NA	NA	NA
**Selection of the reported result**												
5.1 Were the data that produced this result analysed in accordance with a pre-specified analysis plan that was finalized before unblinded outcome data were available for analysis?	YES	YES	YES	YES	YES	YES	YES	YES	YES	YES	YES	YES
Is the numerical result being assessed likely to have been selected, on the basis of the results, from...<br>5.2. ... multiple eligible outcome measurements (e.g. scales, definitions, time points) within the outcome domain?	NO	NO	NO	NO	NO	NO	NO	NO	NO	NO	NO	NO
5.3 ... multiple eligible analyses of the data?	NO	NO	NO	NO	NO	NO	NO	NO	NO	NO	NO	NO

**Risk of bias judgement**	LOW	LOW	LOW	LOW	LOW	LOW	LOW	LOW	LOW	LOW	LOW	LOW
**Optional: What is the predicted direction of bias arising from the randomization process?**	NA	NA	NA	NA	NA	NA	NA	NA	NA	NA	NA	NA
**Overall risk of bias**	LOW	LOW	HIGH	HIGH	LOW	LOW	LOW	LOW	LOW	UNCLEAR	LOW	LOW
**Optional: What is the overall predicted direction of bias for this outcome?**	NA	NA	UNCLEAR	UNCLEAR	NA	NA	NA	NA	NA	UNCLEAR	NA	NA

Abbreviations: NA, not applicable.

Table S13. Input table for ARR analysis

Trial	Duration/Timepoint (weeks)	Arm	Intervention	ARR	N
AFFIRM	104	1	Placebo	0.73	315
AFFIRM	104	2	Natalizumab 300 mg	0.23	627
ASCLEPIOS I	130	1	Teriflunomide 14 mg	0.22	452
ASCLEPIOS I	130	2	Ofatumumab 20 mg	0.11	454
ASCLEPIOS II	130	1	Teriflunomide 14 mg	0.25	469
ASCLEPIOS II	130	2	Ofatumumab 20 mg	0.10	469
CARE-MS I	104	1	IFNB-1a 44 mcg	0.39	187
CARE-MS I	104	2	Alemtuzumab 12 mg	0.18	376
CARE-MS II	104	1	IFNB-1a 44 mcg	0.52	202
CARE-MS II	104	2	Alemtuzumab 12 mg	0.26	426
IMPROVE	16	1	Placebo	0.33*	60
IMPROVE	16	2	IFNB-1a 44 mcg	0.14*	120
OPERA I	96	1	IFNB-1a 44 mcg	0.29	411
OPERA I	96	2	Ocrelizumab 600 mg	0.16	410
OPERA II	96	1	IFNB-1a 44 mcg	0.29	418
OPERA II	96	2	Ocrelizumab 600 mg	0.16	417
OWIMS	48	1	Placebo	0.64¶	100
OWIMS	48	2	IFNB-1a 22 mcg	0.67¶	95
OWIMS	48	3	IFNB-1a 44 mcg	0.60¶	98
PRISMS	104	1	Placebo	0.84¶	187
PRISMS	104	2	IFNB-1a 22 mcg	0.73¶	189
PRISMS	104	3	IFNB-1a 44 mcg	0.68¶	184
TEMSO	108	1	Placebo	0.54	363

TEMSO	108	2	Teriflunomide 7 mg	0.37	365
TEMSO	108	3	Teriflunomide 14 mg	0.37	357
TENERE	96	1	IFNB-1a 44 mcg	0.22	104
TENERE	96	2	Teriflunomide 7 mg	0.41	109
TENERE	96	3	Teriflunomide 14 mg	0.26	111
TOWER	152	1	Placebo	0.50	388
TOWER	152	2	Teriflunomide 7 mg	0.39	407
TOWER	152	3	Teriflunomide 14 mg	0.32	370
ULTIMATE I	96	1	Teriflunomide 14 mg	0.19	274
ULTIMATE I	96	2	Ublituximab 150 mg and 450 mg	0.08	271
ULTIMATE II	96	1	Teriflunomide 14 mg	0.18	272
ULTIMATE II	96	2	Ublituximab 150 mg and 450 mg	0.09	272

Abbreviations: ARR, annualized relapse rate.
*Relapse rate based on 16-week study duration.
¶Calculated based on % of patients who were not relapse free.

Table S14. Input table for CDP-3m analysis

				Reported Data					
				Source 1		Source 2		NMA data	
**Trial**	**Duration/Timepoint (weeks)**	**Arm**	**Intervention**	**Type**	**Value**	**Type**	**Value**	**Log(HR)**	**SE(Log(HR))**
AFFIRM	104	1	Placebo						
AFFIRM	104	2	Natalizumab 300 mg	95% CI Lower Limit	0.43	95% CI Upper Limit	0.77	-0.545	0.149
ASCLEPIOS I	130	1	Teriflunomide 14 mg						
ASCLEPIOS I	130	2	Ofatumumab 20 mg	95% CI Lower Limit	0.45	95% CI Upper Limit	0.96	-0.431	0.193

ASCLEPIOS II	130	1	Teriflunomide 14 mg						
ASCLEPIOS II	130	2	Ofatumumab 20 mg	95% CI Lower Limit	0.45	95% CI Upper Limit	0.97	-0.416	0.196
OPERA I	96	1	IFNB-1a 44 mcg						
OPERA I	96	2	Ocrelizumab 600 mg	95% CI Lower Limit	0.37	95% CI Upper Limit	0.90	-0.562	0.227
OPERA II	96	1	IFNB-1a 44 mcg						
OPERA II	96	2	Ocrelizumab 600 mg	95% CI Lower Limit	0.42	95% CI Upper Limit	0.92	-0.462	0.200
PRISMS	104	1	Placebo						
PRISMS	104	2	IFNB-1a 22 mcg	95% CI Lower Limit	0.48	95% CI Upper Limit	0.98	-0.386	0.182
PRISMS	104	3	IFNB-1a 44 mcg	95% CI Lower Limit	0.43	95% CI Upper Limit	0.91	-0.478	0.191
TEMSO	108	1	Placebo						
TEMSO	108	2	Teriflunomide 7 mg	95% CI Lower Limit	0.56	95% CI Upper Limit	1.05	-0.274	0.160
TEMSO	108	3	Teriflunomide 14 mg	95% CI Lower Limit	0.51	95% CI Upper Limit	0.97	-0.357	0.164
TOWER	152	1	Placebo						
TOWER	152	2	Teriflunomide 7 mg	95% CI Lower Limit	0.68	95% CI Upper Limit	1.35	-0.051	0.175

TOWER	152	3	Teriflunomide 14 mg	95% CI Lower Limit	0.47	95% CI Upper Limit	1	-0.386	0.193
ULTIMATE I and II	96	1	Teriflunomide 14 mg						
ULTIMATE I and II	96	2	Ublituximab 150 mg and 450 mg	95% CI Lower Limit	0.50	95% CI Upper Limit	1.41	-0.174	0.264

Abbreviations: HR, hazard ratio; NMA, network meta-analysis; SE, standard error.

Table S15. Input table for CDP-6m analysis

				**Reported Data**					
				**Source 1**		**Source 2**		**NMA data**	
**Trial**	**Duration/Timepoint (weeks)**	**Arm**	**Intervention**	**Type**	**Value**	**Type**	**Value**	**Log(HR)**	**SE(Log(HR))**
AFFIRM	104	1	Placebo						
AFFIRM	104	2	Natalizumab 300 mg	95% CI Lower Limit	0.33	95% CI Upper Limit	0.64	-0.777	0.169
ASCLEPIOS I	130	1	Teriflunomide 14 mg						
ASCLEPIOS I	130	2	Ofatumumab 20 mg	95% CI Lower Limit	0.40	95% CI Upper Limit	0.93	-0.494	0.215
ASCLEPIOS II	130	1	Teriflunomide 14 mg						
ASCLEPIOS II	130	2	Ofatumumab 20 mg	95% CI Lower Limit	0.49	95% CI Upper Limit	1.17	-0.274	0.222
CARE-MS I	104	1	IFNB-1a 44 mcg						

CARE-MS I	104	2	Alemtuzumab 12 mg	95% CI Lower Limit	0.40	95% CI Upper Limit	1.23	-0.357	0.287
CARE-MS II	104	1	IFNB-1a 44 mcg						
CARE-MS II	104	2	Alemtuzumab 12 mg	95% CI Lower Limit	0.38	95% CI Upper Limit	0.87	-0.545	0.211
OPERA I	96	1	IFNB-1a 44 mcg						
OPERA I	96	2	Ocrelizumab 600 mg	95% CI Lower Limit	0.34	95% CI Upper Limit	0.95	-0.562	0.262
OPERA II	96	1	IFNB-1a 44 mcg						
OPERA II	96	2	Ocrelizumab 600 mg	95% CI Lower Limit	0.40	95% CI Upper Limit	0.98	-0.462	0.229
PRISMS	104	1	Placebo						
PRISMS	104	3	IFNB-1a 44 mcg	95% CI Lower Limit	0.50	95% CI Upper Limit	0.90	-0.401	0.150
TEMSO	108	1	Placebo						
TEMSO	108	3	Teriflunomide 14 mg	95% CI Lower Limit	0.51	95% CI Upper Limit	1.11	-0.285	0.198
TOWER	152	1	Placebo						
TOWER	152	2	Teriflunomide 7 mg	95% CI Lower Limit	0.69	95% CI Upper Limit	1.61	0.053	0.216
TOWER	152	3	Teriflunomide 14 mg	95% CI Lower Limit	0.53	95% CI Upper Limit	1.33	-0.171	0.234

ULTIMATE I and II	96	1	Teriflunomide 14 mg						
ULTIMATE I and II	96	2	Ublituximab 150 mg and 450 mg	95% CI Lower Limit	0.36	95% CI Upper Limit	1.21	-0.416	0.309

Abbreviations: HR, hazard ratio; NMA, network meta-analysis; SE, standard error.

Table S16. Input table for treatment discontinuation analysis

Trial	Duration/Timepoint (weeks)	Arm	Intervention	N patients	N events
AFFIRM	104	1	Placebo	312	46
AFFIRM	104	2	Natalizumab 300 mg	627	76
ASCLEPIOS I	130	1	Teriflunomide 14 mg	462	98
ASCLEPIOS I	130	2	Ofatumumab 20 mg	465	65
ASCLEPIOS II	130	1	Teriflunomide 14 mg	474	102
ASCLEPIOS II	130	2	Ofatumumab 20 mg	481	96
CARE-MS I	104	1	IFNB-1a 44 mcg	187	23
CARE-MS I	104	2	Alemtuzumab 12 mg	376	14
CARE-MS II	104	1	IFNB-1a 44 mcg	202	44
CARE-MS II	104	2	Alemtuzumab	596	39
OPERA I	96	1	IFNB-1a 44 mcg	409	69
OPERA I	96	2	Ocrelizumab 600 mg	408	42
OPERA II	96	1	IFNB-1a 44 mcg	417	97
OPERA II	96	2	Ocrelizumab 600 mg	417	57
PRISMS	104	1	Placebo	187	17
PRISMS	104	2	IFNB-1a 22 mcg	189	22
PRISMS	104	3	IFNB-1a 44 mcg	184	19

TEMSO	108	1	Placebo	363	104
TEMSO	108	2	Teriflunomide 7 mg	365	91
TEMSO	108	3	Teriflunomide 14 mg	358	95
TENERE	115	1	IFNB-1a 44 mcg	101	30
TENERE	115	2	Teriflunomide 7 mg	109	20
TENERE	115	3	Teriflunomide 14 mg	111	22
TOWER	132	1	Placebo	388	125
TOWER	132	2	Teriflunomide 7 mg	407	134
TOWER	132	3	Teriflunomide 14 mg	370	126
ULTIMATE I	96	1	Teriflunomide 14 mg	275	23
ULTIMATE I	96	2	Ublituximab 150 mg and 450 mg	273	34
ULTIMATE II	96	1	Teriflunomide 14 mg	273	34
ULTIMATE II	96	2	Ublituximab 150 mg and 450 mg	272	18

Table S17. SUCRA and ranking probabilities for treatments for ARR outcome

Treatment	SUCRA	Probability of being the best (%)	Mean Rank
ofatumumab	85.4	30.8	2.3
ublituximab	83.9	32.6	2.4
natalizumab	83.7	26.4	2.5
alemtuzumab	73.3	8.6	3.4
ocrelizumab	62.5	1.5	4.4
teriflunomide 14	40.5	0.0	6.4
interferon beta-1a 44	32.2	0.0	7.1
teriflunomide 7	22.5	0.0	8.0
interferon beta-1a 22	14.7	0.0	8.7

placebo	1.4	0.0	9.9

Abbreviations: SUCRA, surface under the cumulative ranking.

Table S18. Direct and indirect estimates of effect for the network of ARR outcome for pairwise inconsistency

Comparison	Direct RR (95% CI)	Indirect RR (95% CI)	Inconsistency factor (IF)	Standard error of IF	Inconsistency p-value
placebo vs interferon beta-1a 44*	1.22 (1.09, 1.37)	2.25 (1.46, 3.46)	0.608	0.224	**0.007**
placebo vs interferon beta-1a 22*	1.09 (0.97, 1.23)	4.06 (2.00, 8.21)	1.303	0.369	**0.000**
placebo vs teriflunomide 14 mg	1.52 (1.23, 1.89)	1.16 (0.66, 2.05)	-0.276	0.306	0.368
placebo vs teriflunomide 7 mg	1.36 (1.21, 1.53)	0.66 (0.43, 1.01)	-0.728	0.223	**0.001**
interferon beta-1a 44 vs interferon beta-1a 22*	0.92 (0.82, 1.04)	0.25 (0.12, 0.51)	-1.303	0.369	**0.000**
interferon beta-1a 44 vs teriflunomide 14 mg	0.89 (0.52, 1.51)	1.16 (0.87, 1.56)	0.276	0.306	0.368
interferon beta-1a 44 vs teriflunomide 7 mg	0.54 (0.36, 0.81)	1.12 (0.95, 1.31)	0.728	0.223	**0.001**
teriflunomide 14 mg vs teriflunomide 7 mg*	0.84 (0.69, 1.02)	3.46 (0.10, 115.39)	1.417	979.019	0.999

Abbreviations: CI, confidence interval; IF, inconsistency factor; RR, rate ratio.

* All the evidence about these contrasts comes from the trials which directly compare them. This is to indicate these comparisons are coherent (i.e., consistent) by definition, because they are informed by multi-arm trials. Significant inconsistency p-value means estimates from direct and indirect comparison are statistically different. p-value for global test of inconsistency = **0.002**.

N.B. Statistical tests of inconsistency have low power and thus typically, p-value < 0.1 is considered as important inconsistency; important inconsistency p-values are bolded and highlighted.

Table S19. Sensitivity analysis using inconsistency model for ARR outcome analysis (n = 15 RCTs)

**ublituximab**					
1.47 (0.73,2.93)	**alemtuzumab**				
0.97 (0.65,1.43)	0.66 (0.36,1.22)	**natalizumab**			
1.29 (0.64,2.58)	0.88 (0.68,1.13)	1.33 (0.71,2.48)	**ocrelizumab**		
1.02 (0.68,1.52)	0.70 (0.36,1.33)	1.06 (0.78,1.44)	0.79 (0.41,1.53)	**ofatumumab**	
**0.66 (0.58,0.75)**	**0.21 (0.11,0.38)**	0.91 (0.80,1.03)	**0.31 (0.27,0.37)**	**0.24 (0.13,0.43)**	**placebo**

Results are RR and their 95% CIs. For column compared to row, RR < 1 means the top-left treatment is better [RR > 1 favors the treatment in the row]. **Bolded** comparisons are statistically significant. Blue highlighted cells are effect estimates for the comparison of all active drugs versus placebo.

Table S20. SUCRA and ranking probabilities for treatments for ARR outcome from sensitivity analysis using inconsistency model

Treatment	SUCRA	Probability of being the best (%)	Mean Rank
alemtuzumab	94.3	70.1	1.5
ocrelizumab	83.1	11.5	2.5
ofatumumab	71.1	6.4	3.6
ublituximab	69.6	9.5	3.7
natalizumab	65.7	2.6	4.1
interferon beta-1a 44	48.3	0.0	5.7
teriflunomide 14	33.3	0.0	7.0
teriflunomide 7	23.4	0.0	7.9
interferon beta-1a 22	10.5	0.0	9.1
placebo	0.7	0.0	9.9

Abbreviations: SUCRA, surface under the cumulative ranking.

Table S21. Sensitivity analysis excluding OWIMS and IMPROVE trials for ARR outcome analysis (n = 13 RCTs)

**ublituximab**					
0.89 (0.52,1.51)	**alemtuzumab**				
0.98 (0.60,1.60)	1.10 (0.71,1.71)	**natalizumab**			
0.78 (0.46,1.32)	0.87 (0.63,1.22)	0.79 (0.51,1.23)	**ocrelizumab**		
1.02 (0.65,1.60)	1.15 (0.72,1.84)	1.04 (0.68,1.59)	1.31 (0.82,2.11)	**ofatumumab**	
**0.31 (0.21,0.46)**	**0.35 (0.25,0.49)**	**0.31 (0.24,0.41)**	**0.40 (0.28,0.57)**	**0.30 (0.22,0.42)**	**placebo**

Results are RR and their 95% CIs. For column compared to row, RR < 1 means the top-left treatment is better [RR > 1 favors the treatment in the row]. **Bolded** comparisons are statistically significant. Blue highlighted cells are effect estimates for the comparison of all active drugs versus placebo.

Table S22. SUCRA and ranking probabilities for treatments for ARR outcome from sensitivity analysis excluding OWIMS and IMPROVE trials

Treatment	SUCRA	Probability of being the best (%)	Mean Rank
ofatumumab	85.2	30.3	2.3

ublituximab	83.7	33.7	2.5
natalizumab	82.1	22.9	2.6
alemtuzumab	74.9	11.1	3.3
ocrelizumab	62.9	2.0	4.3
teriflunomide 14	38.8	0.0	6.5
interferon beta-1a 44	32.8	0.0	7.0
teriflunomide 7	20.6	0.0	8.1
interferon beta-1a 22	18.2	0.0	8.4
placebo	0.8	0.0	9.9

Abbreviations: SUCRA, surface under the cumulative ranking.

Table S23. Sensitivity analysis excluding OWIMS and PRISMS trials for ARR outcome analysis (n = 13 RCTs)

**ublituximab**					
1.39 (0.84,2.29)	**alemtuzumab**				
0.94 (0.64,1.38)	0.68 (0.45,1.02)	**natalizumab**			
1.22 (0.73,2.03)	0.88 (0.68,1.13)	1.29 (0.85,1.96)	**ocrelizumab**		
1.02 (0.69,1.52)	0.73 (0.47,1.14)	1.08 (0.80,1.45)	0.84 (0.53,1.31)	**ofatumumab**	
**0.30 (0.21,0.42)**	**0.21 (0.15,0.31)**	**0.31 (0.27,0.37)**	**0.24 (0.17,0.36)**	**0.29 (0.23,0.37)**	**placebo**

Results are RR and their 95% CIs. For column compared to row, RR < 1 means the top-left treatment is better [RR > 1 favors the treatment in the row]. **Bolded** comparisons are statistically significant. Blue highlighted cells are effect estimates for the comparison of all active drugs versus placebo.

Table S24. SUCRA and ranking probabilities for treatments for ARR outcome from sensitivity analysis excluding OWIMS and PRISMS trials

Treatment	SUCRA	Probability of being the best (%)	Mean Rank
alemtuzumab	95.4	72.3	1.4
ocrelizumab	82.5	13.5	2.4
ofatumumab	68.7	5.4	3.5
ublituximab	67.1	8.1	3.6
natalizumab	59.9	0.8	4.2

interferon beta-1a 44	38.8	0.0	5.9
teriflunomide 14	24.9	0.0	7.0
teriflunomide 7	12.7	0.0	8.0
placebo	0.0	0.0	9.0

Abbreviations: SUCRA, surface under the cumulative ranking.

Table S25. Meta-regression analysis adjusting for follow-up duration for ARR outcome

**placebo**					
**0.37 (0.22,0.62)**	**alemtuzumab**				
**0.31 (0.20,0.50)**	0.85 (0.43,1.70)	**natalizumab**			
**0.42 (0.25,0.68)**	1.13 (0.69,1.87)	1.33 (0.68,2.59)	**ocrelizumab**		
**0.31 (0.19,0.50)**	0.83 (0.42,1.64)	0.97 (0.50,1.88)	0.73 (0.38,1.43)	**ofatumumab**	
**0.35 (0.18,0.67)**	0.95 (0.45,2.02)	1.12 (0.51,2.46)	0.84 (0.40,1.77)	1.15 (0.56,2.37)	**ublituximab**

Results are RR and their 95% CIs. For row compared to column, RR < 1 means the bottom-right treatment is better [RR > 1 favors the treatment in the column]. **Bolded** comparisons are statistically significant. Blue highlighted cells are effect estimates for the comparison of all active drugs versus placebo.

p-value for effect modification: 0.672 (duration of follow-up did not reduce between-study heterogeneity).

Table S26. SUCRA and ranking probabilities for treatments for CDP-3m outcome

Treatment	SUCRA	Probability of being the best (%)	Mean Rank
ocrelizumab	93.9	68.6	1.5
ofatumumab	84.2	21.4	2.3
natalizumab	61.1	2.0	4.1
ublituximab	58.0	7.3	4.4
interferon beta-1a 44	52.2	0.0	4.8
interferon beta-1a 22	41.9	0.6	5.6
teriflunomide 14	38.9	0.0	5.9
teriflunomide 7	18.1	0.0	7.6
placebo	1.7	0.0	8.9

Abbreviations: SUCRA, surface under the cumulative ranking.

Table S27. SUCRA and ranking probabilities for treatments for CDP-6m outcome

Treatment	SUCRA	Probability of being the best (%)	Mean Rank
ocrelizumab	84.4	35.4	2.2
alemtuzumab	82.4	29.0	2.4
natalizumab	74.0	14.4	3.1
ublituximab	63.6	16.0	3.9
ofatumumab	61.8	5.1	4.1
interferon beta-1a 44	40.3	0.0	5.8
teriflunomide 14	26.7	0.0	6.9
placebo	8.6	0.0	8.3
teriflunomide 7	8.3	0.0	8.3

Abbreviations: SUCRA, surface under the cumulative ranking.

Table S28. SUCRA and ranking probabilities for all-cause treatment discontinuation outcome

Treatment	SUCRA	Probability of being the best (%)	Mean Rank
alemtuzumab	99.4	96.7	1.1
ofatumumab	73.7	0.9	3.4
ocrelizumab	65.1	0.1	4.1
natalizumab	64.6	1.3	4.2
ublituximab	52.2	1.1	5.3
teriflunomide 7	49.5	0.0	5.5
teriflunomide 14	39.0	0.0	6.5
placebo	36.7	0.0	6.7
interferon beta-1a 22	11.2	0.0	9.0
interferon beta-1a 44	8.5	0.0	9.2

Abbreviations: SUCRA, surface under the cumulative ranking.

Table S29. Direct and indirect estimates of effect for the network of all-cause treatment discontinuation outcome for pairwise inconsistency

**Comparison**	**Direct**<br><br>**RR (95% CI)**	**Indirect**<br><br>**RR (95% CI)**	**Inconsistency factor (IF)**	**Standard error of IF**	**Inconsistency p-value**
placebo vs interferon beta-1a 44*	0.88<br><br>(0.46, 1.68)	0.66<br><br>(0.41, 1.06)	-0.287	0.406	0.479
placebo vs interferon beta-1a 22*	0.78<br><br>(0.42, 1.46)	0.44<br><br>(0.11, 1.84)	-0.575	0.812	0.479
placebo vs teriflunomide 14 mg	1.01<br><br>(0.83, 1.23)	1.21<br><br>(0.56, 2.60)	0.189	0.408	0.643
placebo vs teriflunomide 7 mg	1.05<br><br>(0.86, 1.28)	1.35<br><br>(0.62, 2.96)	0.245	0.413	0.553
interferon beta-1a 44 vs interferon beta-1a 22*	0.89<br><br>(0.48, 1.63)	1.57<br><br>(0.37, 6.69)	0.575	0.812	0.479
interferon beta-1a 44 vs teriflunomide 14 mg	1.52<br><br>(0.91, 2.53)	1.26<br><br>(0.70, 2.27)	-0.189	0.408	0.643
interferon beta-1a 44 vs teriflunomide 7 mg	1.63<br><br>(0.96, 2.77)	1.28<br><br>(0.71, 2.31)	-0.245	0.413	0.553
teriflunomide 14 mg vs teriflunomide 7 mg*	1.05<br><br>(0.88, 1.25)	1.32<br><br>(0.03, 59.05)	0.231	938.046	0.999

Abbreviations: CI, confidence interval; IF, inconsistency factor; RR, rate ratio.

* All the evidence about these contrasts comes from the trials which directly compare them. This is to indicate these comparisons are coherent (i.e., consistent) by definition, because they are informed by multi-arm trials. Significant inconsistency p-value means estimates from direct and indirect comparison are statistically different. p-value for global test of inconsistency = 0.787.

N.B. Statistical tests of inconsistency have low power and thus typically, p-value < 0.1 is considered as important inconsistency; important inconsistency p-values are bolded and highlighted.

Table S30. NMA results for all-cause treatment discontinuation outcome (excluding CARE-MS I and II) (n = 11 RCTs)

**ublituximab**				
1.10 (0.61,1.99)	**natalizumab**			
1.11 (0.60,2.04)	1.00 (0.55,1.85)	**ocrelizumab**		
1.16 (0.74,1.84)	1.05 (0.64,1.74)	1.05 (0.62,1.76)	**ofatumumab**	
0.91 (0.58,1.42)	0.82 (0.56,1.21)	0.82 (0.51,1.31)	0.78 (0.57,1.06)	**placebo**

Results are RR and their 95% CIs. For column compared to row, RR < 1 means the top-left treatment is better [RR > 1 favours the treatment in the row]. **Bolded** comparisons are statistically significant. Blue highlighted cells are effect estimates for the comparison of all active drugs versus placebo.

Table S31. SUCRA and ranking probabilities for treatment discontinuation outcome (excluding CARE-MS I and II)

Treatment	SUCRA	Probability of being the best (%)	Mean Rank
ofatumumab	83.3	31.5	2.3
ocrelizumab	73.2	29.9	3.1
natalizumab	71.7	25.4	3.3
ublituximab	59.2	11.2	4.3
teriflunomide 7	55.2	1.0	4.6
teriflunomide 14	44.2	0.1	5.5
placebo	40.9	0.1	5.7
interferon beta-1a 22	12.2	0.8	8.0
Interferon beta-1a 44	10.0	0.0	8.2

Abbreviations: SUCRA, surface under the cumulative ranking.

Table S32. Meta-regression analysis adjusting for follow-up duration for all-cause treatment discontinuation outcome

**placebo**					
**0.34 (0.14,0.81)**	**alemtuzumab**				
0.82 (0.49,1.38)	2.4 (0.88,6.56)	**natalizumab**			
0.57 (0.14,2.27)	1.65 (0.67,4.07)	0.69 (0.16,3.04)	**ocrelizumab**		
0.82 (0.49,1.39)	2.41 (0.88,6.60)	1 (0.48,2.09)	1.46 (0.32,6.59)	**ofatumumab**	
0.80 (0.34,1.85)	2.33 (0.70,7.77)	0.97 (0.36,2.61)	1.41 (0.26,7.62)	0.97 (0.33,2.79)	**ublituximab**

Results are RR and their 95% CIs. For row compared to column, RR < 1 means the bottom-right treatment is better [RR > 1 favors the treatment in the column]. **Bolded** comparisons are statistically significant. Blue highlighted cells are effect estimates for the comparison of all active drugs versus placebo.

p-value for effect modification: 0.597 (duration of follow-up did not reduce between-study heterogeneity).

SUPPLEMENTAL FIGURES

Figure S1. Network of treatments for ARR outcome analysis

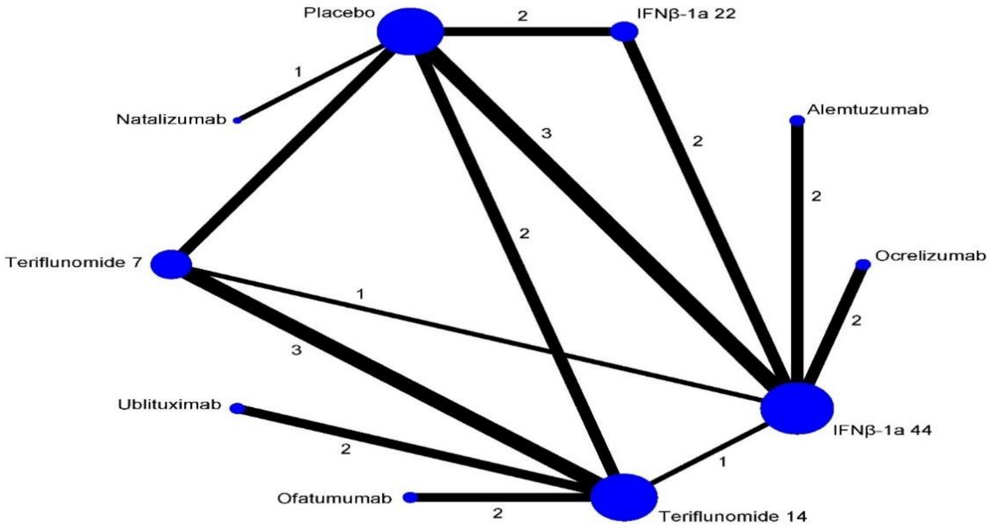

The size of the node (circle) corresponds to the number of patients randomized to that intervention. The thickness of the lines corresponds to the number of studies for each comparison.

Figure S2. Network of treatments for CDP-3m outcome analysis

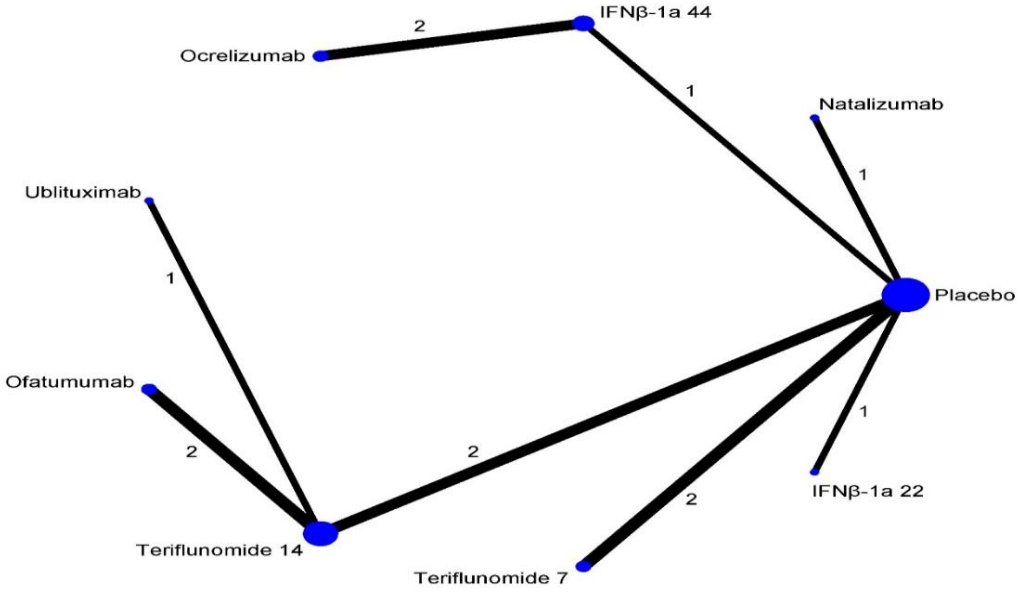

The size of the node (circle) corresponds to the number of patients randomized to that intervention. The thickness of the lines corresponds to the number of studies for each comparison.

Figure S3. Network of treatments for CDP-6m outcome analysis

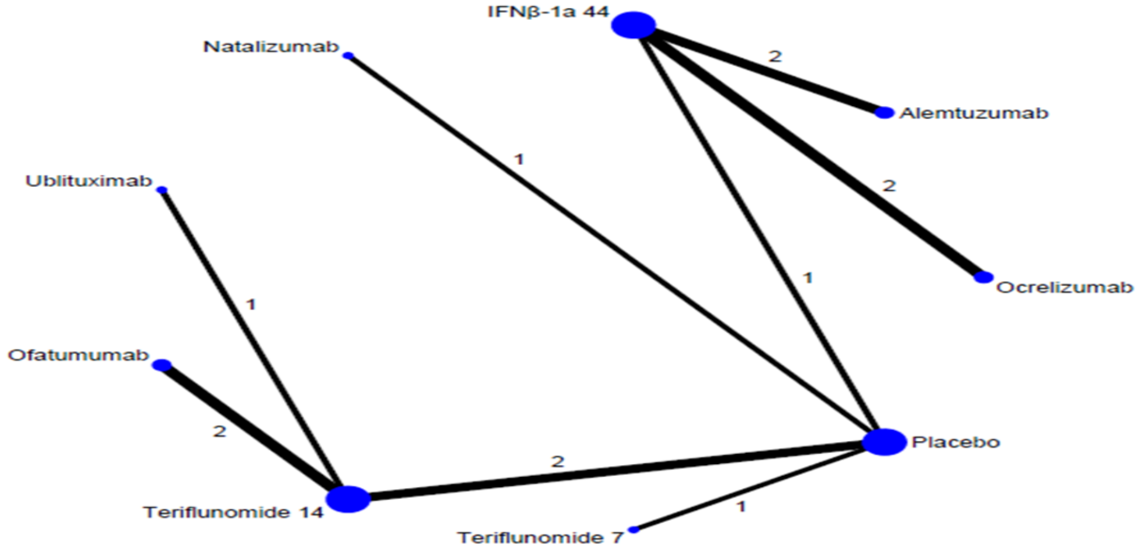

The size of the node (circle) corresponds to the number of patients randomized to that intervention. The thickness of the lines corresponds to the number of studies for each comparison.

Figure S4. Network of treatments for treatment discontinuation outcome analysis

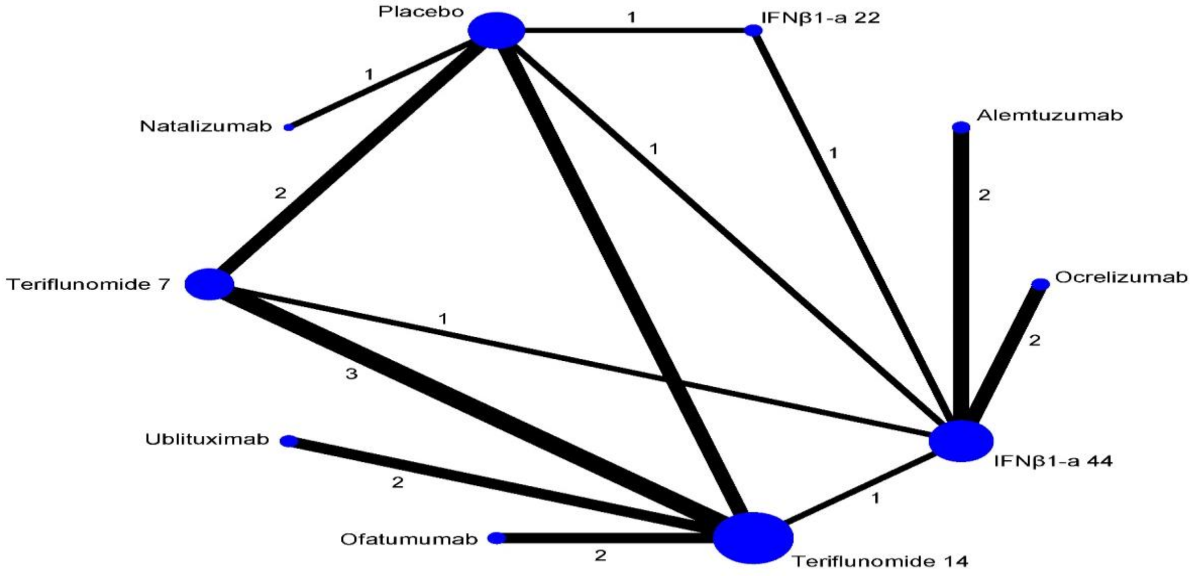

The size of the node (circle) corresponds to the number of patients randomized to that intervention. The thickness of the lines corresponds to the number of studies for each comparison.

REFERENCES

1. Polman CH, O’Connor PW, Havrdova E, Hutchinson M, Kappos L, Miller DH, et al. A Randomized, Placebo-Controlled Trial of Natalizumab for Relapsing Multiple Sclerosis. New England Journal of Medicine. 2006 Mar 2;354(9):899–910.

2. Hauser SL, Bar-Or A, Cohen JA, Comi G, Correale J, Coyle PK, et al. Ofatumumab versus Teriflunomide in Multiple Sclerosis. New England Journal of Medicine. 2020 Aug 6;383(6):546–57.

3. Cohen JA, Coles AJ, Arnold DL, Confavreux C, Fox EJ, Hartung HP, et al. Alemtuzumab versus interferon beta 1a as first-line treatment for patients with relapsing-remitting multiple sclerosis: a randomised controlled phase 3 trial. The Lancet. 2012 Nov;380(9856):1819–28.

4. Coles AJ, Twyman CL, Arnold DL, Cohen JA, Confavreux C, Fox EJ, et al. Alemtuzumab for patients with relapsing multiple sclerosis after disease-modifying therapy: a randomised controlled phase 3 trial. The Lancet. 2012 Nov;380(9856):1829–39.

5. De Stefano N, Curtin F, Stubinski B, Blevins G, Drulovic J, Issard D, et al. Rapid benefits of a new formulation of subcutaneous interferon beta-1a in relapsing—remitting multiple sclerosis. Multiple Sclerosis Journal. 2010 Jul 3;16(7):888–92.

6. De Stefano N, Sormani MP, Stubinski B, Blevins G, Drulovic JS, Issard D, et al. Efficacy and safety of subcutaneous interferon beta-1a in relapsing–remitting multiple sclerosis: Further outcomes from the IMPROVE study. J Neurol Sci. 2012 Jan;312(1–2):97–101.

7. Hauser SL, Bar-Or A, Comi G, Giovannoni G, Hartung HP, Hemmer B, et al. Ocrelizumab versus Interferon Beta-1a in Relapsing Multiple Sclerosis. New England Journal of Medicine. 2017 Jan 19;376(3):221–34.

8. OWIMS Study Group. Evidence of interferon-1a dose response in relapsing-remitting MS The OWIMS Study. Neurology. 1999;53:679–86.

9. PRISMS (Prevention of Relapses and Disability by Interferon -1a Subcutaneously in Multiple Sclerosis) Study Group*. Randomised double-blind placebo-controlled study of interferon-1a in relapsing/remitting multiple sclerosis. THE LANCET. 1998;352:1498–504.

10. O’Connor P, Wolinsky JS, Confavreux C, Comi G, Kappos L, Olsson TP, et al. Randomized Trial of Oral Teriflunomide for Relapsing Multiple Sclerosis. New England Journal of Medicine. 2011 Oct 6;365(14):1293–303.

11. Vermersch P, Czlonkowska A, Grimaldi LM, Confavreux C, Comi G, Kappos L, et al. Teriflunomide versus subcutaneous interferon beta-1a in patients with relapsing multiple sclerosis: a randomised, controlled phase 3 trial. Multiple Sclerosis Journal. 2014 May 14;20(6):705–16.

12. Confavreux C, O'Connor P, Comi G, Freedman MS, Miller AE, Olsson TP, et al. Oral teriflunomide for patients with relapsing multiple sclerosis (TOWER): a randomised, double-blind, placebo-controlled, phase 3 trial. Lancet Neurol. 2014 Mar;13(3):247–56.

13. Steinman L, Fox E, Hartung HP, Alvarez E, Qian P, Wray S, et al. Ublituximab versus Teriflunomide in Relapsing Multiple Sclerosis. New England Journal of Medicine. 2022 Aug 25;387(8):704–14.
